# Restoring Sight: The Journey of AIPL1 from Discovery to Therapy

**DOI:** 10.3390/ijms262412066

**Published:** 2025-12-15

**Authors:** Alima Galieva, Alexander Karabelsky, Alexander D. Egorov

**Affiliations:** Gene Therapy Department, Center for Translational Medicine, Sirius University of Science and Technology, 354340 Sirius, Russia; alima.galieva@gmail.com (A.G.); karabelskiy.av@talantiuspeh.ru (A.K.)

**Keywords:** aryl hydrocarbon receptor interacting protein-like 1, AIPL1, aryl hydrocarbon receptor interacting protein, Leber congenital amaurosis, Leber congenital amaurosis type 4, inherited retinal diseases, adeno-associated virus, gene therapy, photoreceptors, vision

## Abstract

Leber congenital amaurosis (LCA) is a severe inherited retinal disorder manifesting at birth or in early infancy, with a subset of cases linked to mutations in the aryl hydrocarbon receptor-interacting protein-like 1 (*AIPL1*) gene. Initially identified as the disease locus for LCA4, *AIPL1* exhibits a retina-specific expression pattern. Its protein product is a unique member of the FKBP family, distinguished by its specific structural features and specialized functions. A wide spectrum of mutations in *AIPL1* is associated with varying severities of retinal degeneration, implicating diverse pathogenic mechanisms. While the early onset and rapid progression of *AIPL1*-related disorders pose a therapeutic challenge, significant progress in gene therapy has unlocked promising avenues for effective treatment. This comprehensive review summarizes current findings to spark interest and pave the way for further studies in the therapy of *AIPL1*-caused retinal diseases.

## 1. Introduction

Inherited retinal diseases (IRD) are a group of heterogeneous pathologies that can cause serious vision impairments or blindness. Leber congenital amaurosis (LCA) is a severe form of inherited retinal dystrophy with significant genetic diversity. While its reported prevalence varies from 1 in 81,000 [1] to 1 in 30,000 [2] live births, the incidence is often greater in genetically isolated communities or in countries where common consanguineous pairings are frequent [3,4]. Patients with LCA usually manifest at birth with serious vision loss and pendular nystagmus. Electroretinogram (ERG) responses are usually nonrecordable. Other clinical findings may include high hypermetropia, photophobia, oculodigital sign, keratoconus, cataracts, and a variable appearance to the fundus [4].

For now, 28 genes are known to be associated with diverse LCA types: *GUCY2D* (LCA1), *RPE65* (LCA2), *SPATA7* (LCA3), *AIPL1* (LCA4), *LCA5* (LCA5), *RPGRIP1* (LCA6), *CRX* (LCA7), *CRB1* (LCA8), *NMNAT1* (LCA9), *CEP290* (LCA10), *IMPDH1* (LCA11), *RD3* (LCA12), *RDH12* (LCA13), *LRAT* (LCA14), *TULP1* (LCA15), *KCNJ13* (LCA16), *GDF6* (LCA17), *CCT2* (LCA18), *USP45* (LCA19), *PRPH2*, CABP4, *CLUAP1*, *DTHD1*, *IDH3A*, *IFT140*, *IQCB1* or *NPHP5*, *TUBB4B*, and *OTX2* [5,6,7,8]. Clinically significant genetic variations in these genes account for approximately 70–80% of all cases of LCA [7], and new genes linked to LCA are being identified. The mechanisms underlying the pathology that eventually leads to retinal dystrophy can generally be divided into several categories: related to phototransduction (*GUCY2D*, *AIPL1*, *KCNJ13*, *CABP4*), retinoid visual cycle (*RPE65*, *RDH12*, *LRAT*), photoreceptor ciliary transport (*SPATA7*, *LCA5*, *RPGRIP1*, *CEP290*, *TULP1*, *CLUAP1*, *IQCB1*, *IFT140*, *ALMS1*), photoreceptor morphogenesis (*CRX*, *CRB1*, *GDF6*, *PRPH2*), coenzyme NAD biosynthesis (*NMNAT1*), guanine synthesis (*IMPDH1*), protein trafficking (*RD3*), and photoreceptor differentiation (*OTX2*) [5,7].

This review is concentrated on one of the LCA-causing genes, *AIPL1* (Aryl Hydrocarbon Receptor Interacting Protein-Like 1). Leber congenital amaurosis type 4 (LCA4), caused by mutations in *AIPL1*, is one of the most severe early-onset forms of IRD and the cause of congenital loss of vision diagnosed in early childhood [9,10]. AIPL1 is a protein encoded by the *AIPL1* gene, which is primarily expressed in the photoreceptor cells of the retina (rods and cones) and plays an indirect but essential role in the phototransduction cycle and viability of the retina. It functions as a co-chaperone that ensures the proper folding and stability of other critically important proteins. AIPL1 interacts with other proteins, primarily with the phosphodiesterase 6 (PDE6) complex, to ensure the normal phototransduction cascade. Pathogenic mutations in the *AIPL1* gene cause severe IRDs, such as LCA4 and some forms of retinitis pigmentosa (RP).

As the structure of AIPL1 determines functions, there is a substantial necessity to resolve structural ambiguities. The key structural features include the FKBP domain (named after FK506-binding proteins), which binds the isoprenyl moieties of the PDE6 subunits and assists in protein folding; the TPR domain (tetratricopeptide repeat), through which AIPL1 interacts with the chaperone HSP90 and its primary “client”, PDE6; and the intrinsically disordered proline-rich domain (PRD), which regulates chaperone function of AIPL1 [11]. Understanding the structure and function of AIPL1 opens up several promising avenues for gene therapy, as the diseases caused by its mutations are monogenic.

Since *AIPL1* mutations typically result in a loss of function, adding a functional copy of the gene can restore the process of PDE6 maturation and slow down the progression of the disease. Harnessing a relatively safe viral vector (most commonly an adeno-associated virus (AAV)) to deliver a healthy copy of the *AIPL1* gene into the patient’s photoreceptors is a promising therapeutic strategy for the treatment of LCA4: several AAV-based therapies are being investigated at various stages of preclinical and clinical research. One of the AAV2/8-h*AIPL1* gene therapy candidates has progressed to and has been evaluated in a clinical study [12].

Technologies exploiting gene editing, like CRISPR-Cas9, could be used to directly correct the mutation in the *AIPL1* gene within the DNA of retinal cells. This could be the most direct but also technically intricate approach, which is currently in the early stages of development for retinal diseases [13].

The goal of this review is to synthesize current knowledge about the function of the *AIPL1* gene and protein, its structure, and the molecular mechanisms through which its dysfunction causes disease. The primary aim is to link this fundamental knowledge with prospective treatments, the foremost being gene augmentation therapy, which has already reached the clinical study stage and offers hope for treating previously incurable forms of blindness.

## 2. *AIPL1* Gene and Protein

### 2.1. Evolution and Orthologs

The evolutionary path of *AIPL1* includes deep conservation and recent lineage-specific adaptations. Concerning the evolutionary precursor to the *AIPL1* and its paralog *AIP* (aryl hydrocarbon receptor interacting protein), an ancestral *aip* gene already existed in the common ancestor of bilaterian animals [14,15]. Phylogenetic analysis revealed that divergence of *AIP* and *AIPL* emerged from an ancient whole-genome duplication event that occurred early in the evolution of gnathostomes (jawed vertebrates) [12]. The *AIP* clade encompasses homologs across all major vertebrate lineages, including bony fish, amphibians, reptiles, birds, and mammals. Vertebrate-specific *AIPL* proteins fall into two main clades: *AIPL-1* and *AIPL-2*, resulting from the second round of whole genome duplication. Homologs of platypus, avian species, and reptiles are found exclusively in the *AIPL-2* clade, while mammals and amphibians have been shown to lose *AIPL-2* through their evolution [12]. In bony fish, particularly in zebrafish, there are two homologs of the *AIPL1* gene preserved: *aipl1a* (*AIPL-1*) expressed mainly in rods, and *aipl1b* (*AIPL-2*) expressed in cones [15,16].

The critical role of *AIPL1* in vision is conserved across mammals. This is evidently demonstrated by the presence of a nonsense mutation in the *AIPL1* gene of Persian cats, which causes LCA [17]. This mutation leads to a truncated, non-functional protein, which, along with the existence of other animal models of LCA4, confirms that the essential function of AIPL1 has been preserved through evolution.

Human AIPL1 includes a C-terminal PRD that is exclusive to primates [14]. This particular part of the protein has evolved relatively recently, after the divergence of primates from other mammalian lineages. A recent study suggests that this domain is largely dispensable for the protein’s core chaperone function, and variants within it are typically not disease-causing, with one specific exception linked to a dominant form of cone-rod dystrophy [18]. Evolutionary information is crucial for a complete understanding of AIPL1’s function and its role in disease.

### 2.2. Gene Structure and Transcript Variants

The initial identification and chromosomal mapping of the *AIPL1* gene were driven by the analysis of retinal-expressed sequences. Originally, two expressed sequence tags (EST) designed for the retinal/pineal-expressed EST clusters were mapped to 17p13.3 near a retinitis pigmentosa (RP13) candidate region [19]. Later, it was determined that the cDNAs of these two EST clusters overlap and represent coding sequences (CDS) of a single gene. This gene was designated as aryl hydrocarbon-interacting protein-like-1 (*AIPL1*) due to high sequence similarity to human aryl hydrocarbon-interacting protein (*AIP*, also known as HBV X-associated protein 2, *XAP2*, or *ARA9*) [20]. Then, two groups simultaneously attempted to clarify the 17p13.3 chromosome region structure based on known ESTs, but McHale and colleagues failed to map *AIPL1* at this region [21], while Sohocki and colleagues refined *AIPL1* localization to 17p13.1 and confirmed it by fluorescence in situ hybridization. They also demonstrated that the second EST, initially appearing in association with *AIPL1* expression, was a result of transcripts bypassing the first polyadenylation signal, which led to the formation of a 3′-untranslated region (UTR) longer than the first EST by 709 nt [22].

**Figure 1:**
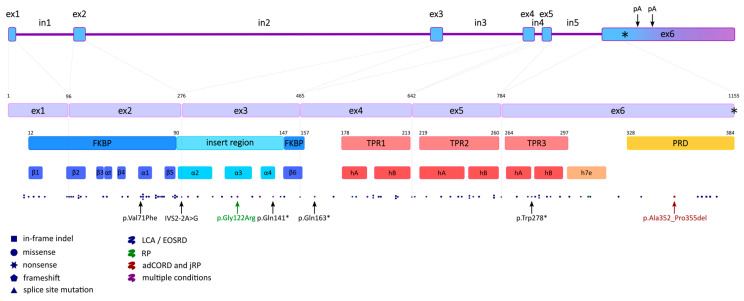
*AIPL1* gene, CDS, and polypeptide annotation. The first upper line of blocks represents the intron-exonic organization of the *AIPL1* gene (asterisk indicates stop codon position, arrows—polyadenylation sites); the second—splice junctions in CDS with nucleotide positions specified [28]; the third—protein domains with polypeptide positions stated; the fourth—secondary structures; and the dotted line at the bottom is the distribution of pathogenic and likely pathogenic mutations, with the dot key indicating the mutation type and the colour—the diseases associated with these alleles. The 5 most frequent mutations (black), the p.Gly122Arg mutation associated with the mildest condition (RP if homozygous) (dark green), and the p.Ala352_Pro355del mutation associated with autosomal dominant cone-rod dystrophy (adCORD) and juvenile RP (jRP) (dark red) are highlighted with arrows; asterisks (*) indicate the occurrence of a stop codon. FKBP domain annotation (12–157) based on [29]; the insert region encompasses residues 90 to 147 [30], three TPRs: TPR1 (178–213), TPR2 (219–260), and TPR3 (264–297) [26], and a crystal structure and a primate-specific C-terminal PRD (328–384) [29,31].

Human *AIPL1* is a protein-coding gene spanning 11,384 base pairs on the current chromosome location 17p13.2 (6,423,738…6,435,121 positions of the GRCh38.p14 genome assembly) encoded on the complementary strand [23]. The length of the *AIPL1* transcripts is 1538 and 2247 nt; they both share 6 identical exons with CDS and differ only in the length of the 3′ UTR, which is the result of the longer transcript bypassing the polyadenylation signal [22] (Figure 1). *AIPL1* isoforms with in-frame exon skipping have been shown to occur normally in the retina by RT-PCR of the healthy human neuroretinal eye tissue [24] and RNA-seq data analyses of three retina-derived datasets [25], but they are far less represented than full-length processed transcripts. The transcript abundance was estimated to be 87% for the full-length transcript and 9.7% and 3.4% for the ΔEx3 and ΔEx2 transcripts, respectively. Skipping of exon 4 (ΔEx4) appears to be a very rare event and was detected in only one of the three independent datasets with the greatest coverage [26]. Alternative splicing of *AIPL1* was shown to be conserved across mammals, and it was hypothesized that certain splice variants might be functionally important and each might have a specific localization, fulfil individual functions, and even possess mutually exclusive interaction ability [27], although no experimental evidence supporting these assumptions was found.

### 2.3. Expression Pattern

Whereas *AIP* is ubiquitously expressed, acting as a co-chaperone with HSP90 in the maturation of aryl hydrocarbon receptor (AhR) and other nuclear receptors [32], *AIPL1*, in contrast, has been shown to be expressed only in photoreceptors and pineal glands in rats and mice with in situ hybridization experiments [22]. In human tissues, Northern blot hybridization identified mRNA molecules of the predicted sizes in total retinal RNA, and a much weaker signal was detected in skeletal muscle and heart after very long exposure, but these transcripts differ in size from the retinal mRNAs, so these results were considered as cross-hybridization [22]. Single-cell RNA sequencing of retinal organoids made from patient-derived induced pluripotent stem cells (iPSCs) testified that *AIPL1* transcripts are present both in rods and cones, with rod expression being slightly higher [33], while the story of AIPL1 protein expression evaluation was way more entangled. Immunofluorescence confocal microscopy demonstrated that in adult human retinas, AIPL1 is present in rod photoreceptor cells of the peripheral and central human retina, but not in cones [34]. Further study showed that AIPL1 retinal expression changes during the ontogenesis: AIPL1 protein was detected by 11.8 fetal weeks in the central fetal retina, then with continued development, its expression spread gradually towards the periphery of the retina [35]. AIPL1 was shown to be expressed in the rod as well as in the L/M and S cone photoreceptors of the developing human retina [35,36]. But at some time between 40 weeks of development and adulthood, its expression is downregulated in cone photoreceptors. In the developing rod and cone photoreceptors, AIPL1 is located in the presumptive outer and inner segments as well as the photoreceptor cell bodies. In the adult retina, AIPL1 is present only in the inner segments, cell bodies, and spherules of the rod photoreceptors. AIPL1 is critically required during retinal development, as its spatial and temporal pattern of expression precisely coincides with photoreceptor differentiation [35,37], and patients with LCA4 suffer from early onset of vision loss [22]. In another work with macaque retina samples, it has been specified that after birth, progressive loss of AIPL1 in cones happens, with the inner segment losing it first and synapses last. By the end of year 1, AIPL1 staining in cones was dramatically reduced or absent [38]. In human LCA4 patients, severe rod photoreceptor degeneration can occur, and the surviving non-functional photoreceptors are more cone-like, although these cone-like photoreceptors appear to be morphologically abnormal and non-functional [39], which reinforces the theory that rods are more sensitive to *AIPL1* presence than cones. In support of the overriding role of *AIPL1* on rods, some heterozygous carriers of a mutation in *AIPL1* exhibited reduced rod-dependent visual response, likely due to haploinsufficiency, while having a normal cone response [40]. Also, some children with a mutant *AIPL1* allele have been diagnosed with juvenile RP, a rod-specific disease [41]. Comparative analysis of *AIPL1* orthologs provides additional support to the theory about the optionality of AIPL1 for adult cones. It was revealed that in *Danio rerio*, rod *Aipl1a* is more similar to human *AIPL1* than to cone *Aipl1b* [16], which might suggest that the only remaining *AIPL1* ortholog in humans might be more indispensable for rods than for cones, at least in adulthood. On the other hand, it raises the question: which protein performs the function of the lost cone paralog and hints that human *AIPL1* still might play a part in cones at the early stages of retina development.

Finally, more detailed contemplation showed that AIPL1 is also expressed in adult human cones, but at extremely reduced levels [42]. With transgenic mouse models that express human AIPL1 solely in rod photoreceptor cells, a slower rate of cone degeneration has been demonstrated if viable rods are present, while complete loss of the cone electrical response was observed. In this model, cone cells possess disorganized outer segments, which may compromise their viability and contribute to their eventual degeneration. The PDE6 protein complex is absent in these surviving cones, while *PDE6* subunit transcripts are detected, which indicates that AIPL1 may be directly or laterally involved in the regulation of not only rod PDE6α but also cone PDE6α′ subunits. Possible mechanisms of prolonged survival of cone photoreceptors are the “bystander effect” caused by the presence of rod-derived cone viability factor (RdCVF) in the retina [43,44] and/or higher tolerance of cones to Ca^2+^ levels (since the endogenous levels of cGMP and resulting calcium levels are normally higher in cones compared with rod photoreceptors) [45,46]. These results indicate that not only is AIPL1 necessary for the function and survival of cones at some stages of retina development, but also that rods provide some form of protection to cone photoreceptors that prolongs their survival when AIPL1 is absent in cones [42,47].

There is an implication for the therapy of retinal dystrophy: if the cone cells in the central retina of LCA patients survive for a longer period of time [39], then the window of gene therapy application could possibly be extended, allowing for the preservation of cone vision [48].

According to the meta-analysis of the cancer dataset, a down-regulation in the expression of *AIPL1* in different tumours was observed [49]. However, in more detailed published data of this author, the fold change in AIPL1 expression between normal and cancer tissues did not exceed 1.5 in any of the considered datasets [50]. In another work, it has been shown with single-cell RNA sequencing that in retinoblastomas formed from retinal organoids made from patient-derived iPSCs, the *AIPL1* expression level in each residual cell type was comparable with healthy organoid rods, while in healthy organoids, *AIPL1* expression was observed only in photoreceptor cells. Although it is worth mentioning that expression of another photoreceptor-specific gene, *PDE6H*, was at a high level in all retinoblastoma residual cell types [33].

### 2.4. Protein Structure

**Figure 2:**
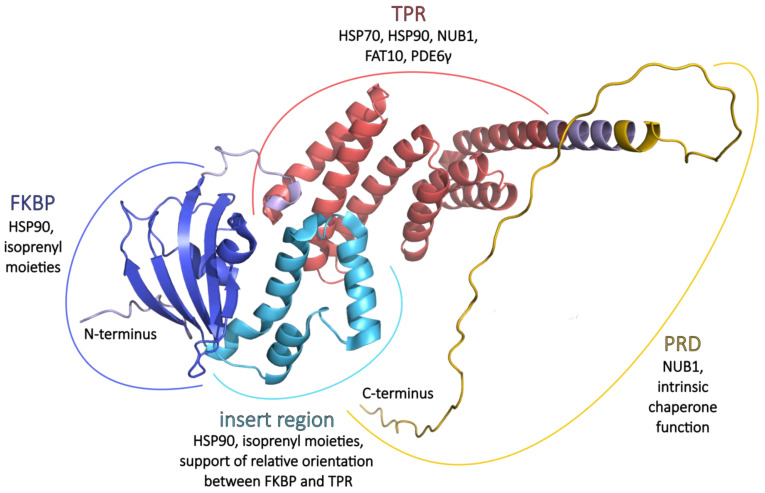
*AIPL1* three-dimensional structure and the main functions. FKBP (blue) and insert region (cyan) architecture based on crystal structure PDB ID 5U9A [30]. TPR (red) organization based on crystal structure PDB ID 6PX0 [55]. The N-terminus, connecting loop between FKBP and TPR domains, C-terminal helix extension (pale purple), and PRD (yellow) were modelled with AlphaFold Server [56]. Figure modified from [57].

AIPL1 is a 384-amino acid protein with a predicted molecular weight of about 43.9 kDa [22], and the pI is 5.57 due to a negative net charge [11]. Human AIPL1 shares 49% amino acid sequence identity and 69% similarity with the human AIP [22]. Despite the high sequence similarity, AIPL1 has vast structural differences from AIP, which will be discussed further, and, unlike AIP, AIPL1 has been shown to exhibit chaperone activity and prevent the aggregation of non-native proteins [11]. In particular, AIPL1 is validated as an obligate chaperone of PDE6 complex subunits, while AIP fails to appreciably bind the isoprenyl (farnesyl and geranylgeranyl) moieties and to chaperone PDE6 [51]. AIPL1 protein consists of an N-terminal FK506-binding protein (FKBP)-like domain (also known as prolyl peptidyl isomerase (PPIase) or immunophilin domain), three tetratricopeptide repeats (TPRs), and a primate-specific C-terminal PRD [29,31,52,53] (Figure 1 and Figure 2). AIPL1 features two hydrophobic patches located near the N-terminus and the linker region between the FKBP-like domain and the TPR domain. In contrast, the C-terminal PRD is hydrophilic. The FKBP-like domain and the proline-rich region contain most of the negatively charged amino acid residues, which contribute to the total negative net charge of AIPL1 [11]. A strongly negative net charge is a hallmark of many well-characterized chaperones, such as HSP90 and HSP70, as it enhances protein solubility and prevents aberrant aggregation [54]. Therefore, distinct physicochemical properties of AIPL1, such as the presence of negatively charged surfaces, contribute to its specialized co-chaperone function within the photoreceptor cells.

Despite the fact that the structure of the whole human AIPL1 protein has not been deciphered to this day, the atomic structure of individual FKBP and TPR domains has been resolved and deposited at Protein Data Bank, PDB ID 5U9A [30] and 6PX0 [55] (Figure 2).

Features of AIPL1-specific amino acid and splice mutations and their role in protein structure and function are compiled and listed in Supplementary Table S1.

#### 2.4.1. FKBP

The AIPL1 FKBP-like domain has the typical FKBP fold consisting of a six-stranded β sheet that forms a half β-barrel around a short α-helix (α1), creating a hydrophobic cavity [30,58]. At the same time, FKBP domains of AIPL1 and AIP are untypical in several aspects: they do not bind immunosuppressant drug FK506 [58], have no PPIase activity [11], and they have long “insert” regions of equivalent lengths (57 aa) linking the last two β strands in the FKBP-like domain that replace the hairpin loop of classic FKBPs and that cover the conical half β-barrel and the hydrophobic cavity [30,58]. The insert regions of AIP and AIPL1 exhibit significant structural divergence. In AIP, the insert regions consist of a 19-residue-long helical segment followed by a mostly random coil structure and an α-helix [58]. In contrast, the insert region in AIPL1 (residues 90–146) is well structured and comprises three consecutive α-helices (α2, α3, and α4) connected by short loops [30]. Further differences between the AIP and AIPL1 FKBP-like domains include the absence of an N-terminal α-helix in AIPL1, which is thought to structurally stabilize the AIP FKBP-like fold, and a loop between β4 and α1 that adopts a ‘looped-out’ conformation in AIPL1 but a ‘looped-in’ conformation in AIP. In the latter, a conserved critical hinge residue, Trp72, is either flipped in or out, respectively [30]. This cavity is formed by hydrophobic residues Phe35, Phe37, Met59, Ile61, Phe87, Cys89, Phe149, and Ile151 in the half β-barrel; residues Val71, Trp72, Leu75, and Leu76 of α1; and Val96, Tyr97, Leu100, Leu104, and Met107 of α2 (insert region). The closed conformation of the β4-α1 loop at the opening of the hydrophobic pocket in AIPL1-FKBP dominates in solution [30], but in complex with HSP90, it changes to an open conformation [59]. Multi-body refinement of the cryo-EM (cryogenic electron microscopy) data indicated large swing-like movements of the AIPL1-FKBP domain [59].

The AIPL1 FKBP-like domain takes part in interaction with HSP90 [29,59], unlike the complex of HSP90 with AIP, where no direct interaction between AIP-FKBP and HSP90 was detected [60]. The α3-helix of the insert region of AIPL1 is located in close proximity to HSP90, which agrees with a moderate contribution of the insert region to the AIPL1-HSP90 interface [61].

The expression of TPR  +  PRD or the TPR domain alone has been shown not to restore cGMP levels to those seen with full-length wild-type AIPL1, confirming that the FKBP domain is absolutely essential for the function of the PDE6 complex, which mediates phototransduction [18]. The FKBP domain of AIPL1 interacts with the farnesyl modifications of the α subunits of cGMP phosphodiesterase 6 (PDE6α) in contradistinction to the FKBP domain of AIP [51], again indicating that the AIPL1–FKBP domain is uniquely specialized [30]. The FKBP-like domain is considered the only structure in AIPL1 capable of binding farnesylated-Cys [62]. Further structural studies showed that the PDE6 subunits’ prenylation is essential for their binding to AIPL1 [30,62], but these results were refuted later [61]. In this latter work, it has been revealed that revoked prenylation of PDE6 subunits (both geranylgeranyl-less PDE6α′ in an in vitro expression system and farnesyl-less PDE6α mouse model) does not significantly impair AIPL1-binding capacity, PDE6 trafficking, or its functionality. In addition, steric occlusion of the AIPL1 prenyl-binding site (in an Ile61Phe/Ile151Phe double AIPL1 mutant) has been shown not to impair AIPL1’s ability to co-chaperone PDE6α′, which altogether indicates that the prenyl-binding feature of AIPL1 is not mandatory. Putatively, AIPL1 gained and retained this facultative prenyl-binding capacity as a slightly increased affinity of the HSP90/AIPL1 complex for the prenylated client, and a more stable ternary complex resulted in an accelerated rate of PDE6 maturation [61]. Both the insert region, particularly the α2 side chains [30,62], and the core domain of AIPL1-FKBP contribute to the binding of the prenyl moiety, with the β4-α1 loop of the core domain playing a crucial role [30], while the Trp72 residue modulates the access of lipid moieties [30,62]. These AIPL1 regions were also shown to be important not only for binding of lipid structures but also for chaperoning of PDE6α′ in the ternary complex of HSP90 dimer, AIPL1, and PDE6 catalytic subunits [61].

The α3-helix of the FKBP insert region was proposed to be important for the dynamics and interdomain interactions of AIPL1. The deletion of this region (AIPL1-Δα3) leads to physical separation of the FKBP and TPR domains [55]. There was an interesting suggestion that the insert region in AIPL1 might be engaged in steric clashes with the TPR domain to provide the required relative orientation between the FKBP and TPR domains [62].

Nuclear magnetic resonance (NMR) studies demonstrated that the FKBP-like domain of human AIPL1 can attain the native fold in the absence of the TPR domain and successfully bind a farnesyl moiety [55]. This was corroborated by another study showing that the AIPL1 FKBP-like domain binds a farnesyl probe comparably to full-length AIPL1 in the absence of the TPR domain, which itself was unable to bind the farnesyl probe and did not alter the affinity of the FKBP-like domain for the probe [62]. However, farnesyl-binding by the FKBP-like domain alone is insufficient for AIPL1 to fulfil its role in modulating PDE6 subunit stability and activity, which also requires a functional interaction with HSP90, which is mediated by the presence of the TPR domain [29].

Notably, whilst the FKBP-like domain of AIPL1 is considered to bind the isoprenoid moieties indiscriminately [30], the function of only PDE6 is affected in the *Aipl*1 knockout and knockdown mouse models [63], which is surprising given the multitude of isoprenylated proteins in the phototransduction cascade. This specificity suggests that features beyond the interaction of AIPL1 with the PDE6 isoprenoid groups must facilitate the specific recruitment of PDE6 to HSP90 by AIPL1 [55,57]. The TPR domain of AIPL1, which is able to interact with the Pγ subunit, seems to fulfil this function [63].

Atomic structures of the human AIPL1 FKBP domain alone and in complex with S-farnesyl-l-cysteine methyl ester (FC) and geranylgeranyl pyrophosphate (GGpp) are deposited at the Protein Data Bank, PDB ID 5U9A, 5U9I, and 5U9J, respectively [30].

#### 2.4.2. TPR

The TPR domain of AIPL1 consists of three consecutive tetratricopeptide motifs, adopts its typical fold, and structurally resembles the TPR domain of AIP. Each TPR motif consists of a pair of anti-parallel α-helices. Three such motifs (1–2, 3–4, and 5–6) form a series of six anti-parallel α-helices [55] interconnected by short loops [64] followed by a seventh α-helix, which altogether forms a right-handed helical array and creates a positively charged groove that serves as a binding surface for TPR domain interaction partners [55].

The AIPL1 TPR domain serves as a binding site for cytosolic HSP90 [31], the regulatory rod PDE6γ, and cone PDE6γ’ subunits [52,55]. These interactions were demonstrated to be specific to the TPR domain of AIPL1 since they do not occur with AIP. A conserved C-terminal segment of the Pγ subunit binds the AIPL1 TPR domain but not the FKBP-like domain. Molecular modelling further indicated that the C-terminal 25 residues of the rod Pγ (63–87 residues) encompass most, if not all, of the contact with the TPR domain, which overlaps the HSP90-binding site. This suggests that the Pγ subunits and HSP90 bind TPR in a mutually exclusive manner. The role of this phenomenon was explained by a model [57] in which the interaction of the inhibitory subunits with the AIPL1 TPR domain imparts specificity toward the PDE6 client, whereas the FKBP-like domain binds isoprenyl moieties indiscriminately, and the HSP90 acceptor site becomes bound competitively by TPR domain co-chaperones. Likewise, the AIPL1 TPR domain partner, HSP90 or Pγ, might shift the equilibrium toward the open conformation of AIPL1–FKBP, which is more accessible for binding of prenyl moieties than closed, thereby promoting formation of the chaperone–PDE6 complex and enabling the expression of functional PDE6 [53,55,57].

TPR consensus residues required for the antiparallel packing of adjacent α-helices in the TPR motifs and residues involved in tight electrostatic interactions with the C-terminal MEEVD TPR acceptor sites of HSP90 are conserved in AIPL1, which is highlighted by its disruption by LCA-associated mutations, which significantly impair its interaction with HSP90 [18,29,31]. These mutations span the following positions and variants of AIPL1: p.Tyr194*, p.Ala197Pro, p.Ser198Phe, p.Ile206Asn, p.Trp222* [18], p.Cys239Arg [31], p.Glu245* [18], p.Gly262_Ala275del [29], p.Lys265Ala [31], p.Arg270His [18], p.Trp278* [29,31], p.Glu282_Ala283dup [29], p.Leu293Pro [18]. The indispensability of the TPR for AIPL1 structure and function is even more underlined by the fact that sequence conservation of the protein is highest across the three AIPL1 TPR motifs [14]. The recent study showed that the TPR alone in the absence of FKBP and PRD can bind HSP90 subunits significantly better than native AIPL1, AIPL1 lacking the PRD, or AIPL1 lacking the FKBP-like domain [18], highlighting the decisive role of TPR in HSP90-inding, while other AIPL1 regions might play a rather regulatory role.

In addition to the role of the core TPR domain contacts in mediating the interaction of AIPL1 with the HSP90, extra requirements for this interaction have been investigated. It appears that AIPL1-TPR α-helical extension (H7e) is involved in interactions with the C-terminal domain of HSP90 [31,59,61]. Interestingly, truncation of the H7e at topologically equivalent residues in FKBP51 and FKBP52 abrogates HSP90 interaction, while removal of the α-helical extension C-terminal to the core TPR domain of human AIPL1 by truncation at the topologically equivalent residue (Glu317) only attenuates the interaction of AIPL1 with HSP90 and HSP70. Similarly, the truncation of the 12 C-terminal residues in mouse AIPL1 (AIPL1 1-316) did not abrogate the HSP90-binding but moderately reduced the affinity for HSP90 as measured by bio-layer interferometry (BLI) [61]. Despite this modest effect on HSP90 interaction, this region is critical for the ability of AIPL1 to chaperone PDE6 in a heterologous assay for the cone PDE6α′ function. This suggests that in addition to the core TPR domain contacts, residues within the H7e may be important for functional chaperone complex assembly. This is notable, as several residues thought to mediate contact of the α-helical extension of FKBP51 with HSP90 are absent or not conserved in mouse or human AIPL1 [57]. Hence, AIPL1 would make fewer contacts with the HSP90 C-terminal domain, and the complex of HSP90 with AIPL1 is significantly weaker than that with FKBP51 [61]. But as the TPR acceptor site of HSP90 can competitively bind a variety of TPR domain co-chaperones, it has been proposed that close contacts with the α3 helix of the FKBP domain may contribute to the specificity of the interaction of AIPL1 with HSP90 [57]. Therefore, H7e may serve just as a client recognition element in AIPL1 [61], as supported by its high mobility within the chaperone complex [59].

Crystal structure of the human AIPL1 TPR domain is deposited at the Protein Data Bank, PDB ID 6PX0 [55].

#### 2.4.3. PRD

PRD assumes an extended monomeric random coil conformation at the last 56 amino acids of the AIPL1 protein, with an abundance of proline and negatively charged amino acid residues [11]. This motif is absent in the majority of vertebrate AIPL1 proteins and shows considerable sequence variation [14].

The PRD of AIPL1 demonstrates unique evolutionary dynamics. While human and chimpanzee sequences are identical in length, the PRD in other primates shows length variations that do not correlate with their evolutionary divergence. This suggests the region is prone to expansion and contraction, likely through replication slippage facilitated by its repetitive XXPP sequence. Notably, despite the PRD’s overall low sequence conservation compared to the stable TPR domains, a subset of residues is strictly preserved, hinting at a critical, conserved function in primates [14].

PRD was first predicted to be intrinsically disordered [65], and then, confirming experimental data was obtained [52]. Due to its extended conformation, PRD appears not to interact with the FKBP-like and TPR domains. Structural studies revealed that the truncation of PRD, but not mutations within it, reduces the molecule’s radius of gyration and maximum dimension. AIPL1 secondary structure and stability are not affected by the pathogenic p.Ala352_Pro355del mutation in PRD, and they have been shown to cause little or no changes in hAIPL1-binding to known partners PDE6α and HSP90 [52]. Studies have confirmed that the deletion of the PRD does not alter the structural integrity or thermal stability of AIPL1; however, it changes the ability of AIPL1 to bind non-native proteins, suppresses the thermal aggregation of citrate synthase, and protects it from thermal inactivation, from which it was concluded that PRD contributes to intrinsic chaperone function [11]. Additionally, it has been shown that the interaction of AIPL1 with the inhibitory PDE6γ subunit might be altered by the p.Ala352_Pro355del mutation [52], which indicates a regulatory role of PRD. Also, deletion of PRD modestly increased the affinity of hAIPL1 for HSP90, as surface plasmon resonance (SPR) assay indicated, suggesting that the PRD may play a role as a negative regulator of the AIPL1-HSP90 interaction [11]. Summarizing the last two facts and a model in which HSP90 and Pγ compete for binding with the AIPL1 TPR domain, it can be assumed that PRD might take part in these regulative interactions along with TPR.

But a recent comprehensive experimental report seems to disagree with previous data: their enzyme-linked immunosorbent assay (ELISA) and cGMP test results convincingly indicate that the PRD is completely dispensable for both the interaction with HSP90 and the modulation of PDE6 activity via an indirect assay of cGMP levels [15]. Deletion of the PRD does not affect the interaction of AIPL1 with the HSP90 C-terminal peptide TSRMEVEED or with a farnesylated and carboxymethylated C-terminal PDE6α peptide [15]. Also, most of the disease-associated mutations in the PRD or the absence of the whole PRD had no effect on the interaction with HSP90 [15]. This enigmatic contradiction might be caused by the different sensitivity of methods used in diverse studies, and the real role of PRD of AIPL1 still waits to be determined. Since the p.Ala352_Pro355del mutation in the PRD is associated with autosomal dominant CORD and RP in patients [28,66] and its pathogenicity was supported by the mouse model [66]. The function of the AIPL1 PRD might include alternative interactors and a mechanism that remains undiscovered.

## 3. Interacting Partners

Beyond the intensively studied AIPL1 interactants, which will be discussed further (Table 1), the literature also describes putative interactors predicted from indirect evidence, as well as others that are mentioned sporadically. For example, the influence of AIPL1 on RetGC1 level in cone photoreceptors was shown in three publications in animal models, although a comprehensive analysis was performed, including retina immunohistochemistry and retinal extracts immunoblotting [16,66,67]. Interaction with cone Pγ’ (encoded by the *PDE6H* gene) was not shown directly, but might be concluded from the conservation with rod Pγ (encoded by the *PDE6G* gene) C-terminal site, which was shown to interact with AIPL1 [55]. p23 co-chaperone was proposed as a potential contributor in the maturation of PDE6 catalytic subunits in complex with HSP90 and AIPL1; however, no direct interaction with AIPL1 was suggested [59]. In unpublished conference abstracts of Bhupesh Parise, Markus N. Preising, and B. Lorenz, there was an indication of a set of AIPL1 interactants detected by yeast two-hybrid assays (CENP-F, DHX32, C15ORF17, TF, PBXIP1, NDUFS6, BEX4, AK2, ITGB4, SAP30, ZFP106), none of which were reported in any other publications (results obtained by direct request to authors), except for CENP-F, confirmed to be an interactor of AIPL1 in another work of these colleagues [68]. Also, AIPL1 was once noticed co-localizing with EB1 and EB3 proteins in the connecting cilia in human retinal photoreceptors [69]. Bovine AIPL1 was shown to interact with apoptosis regulator Aven in the yeast two-hybrid system, and this binding was verified with immunoprecipitation and immunohistochemistry [70], but we failed to find any confirmation of this interaction with human AIPL1.

### 3.1. HSP70 and HSP90

Full-length human AIPL1 has been shown to interact with HSP90 and HSP70 by yeast two-hybrid analysis, and these interactions were validated by in vitro biochemical assays [29,31] and structural studies [61]. AIPL1 interaction with HSP70 appears to be weaker than with HSP90, but the same amino acid residues take part in this interaction [31].

AIPL1 preferentially interacts with the HSP90 dimer in its closed ATP-bound state at a 1:2 stoichiometry. This specific HSP90/AIPL1 complex has been shown to be essential for the maturation of PDE6 in transfected HEK293T cells [61]. Structural studies indicate that the FKBP domain, particularly the β2-β3 loop, plays a key role in HSP90-binding, while the TPR domain interacts with the HSP90 C-terminal domain. The N-terminal region of AIPL1 extends into the HSP90 lumen in a manner that was previously observed for HSP90 clients. N-terminal acetylation of AIPL1 is likely to stabilize this interaction through hydrogen bonding [59].

FKBP-like and TPR domains of AIPL1 can fold independently to acquire the native conformation [30,55,62,76]. But it has been reported that the AIPL1 FKBP-like domain alone cannot interact with HSP90 in the absence of the TPR domain. For example, the LCA-associated patient mutation, p.Glu163*, resulting in the loss of the entire TPR domain and PRD, completely abolished the interaction of AIPL1 with HSP90, emphasizing the critical role of the TPR domain in HSP90 interaction [29]. At the same time, the FKBP-like domain was shown to be severely important for stable ternary chaperone complex formation: patient-associated missense mutations and in-frame deletions in the FKBP-like domain diminished the interaction of AIPL1 with HSP90 and impacted rod PDE6 activity [18,29]. Similarly, replacement of the α3 helix in the AIPL1 FKBP-like unique insert region with flexible glycine linker residues modestly affected the interaction with HSP90 but critically impacted the activity of cone PDE6α′ [61].

Interestingly, there also might be a p23 molecule in complex with HSP90 dimer with AIPL1, which does not significantly change the affinity of HSP90 binding to AIPL1. Thus, p23 was suggested as a potential part of the chaperone–client complex in the maturation of PDE6 [59].

### 3.2. NUB1 and FAT10

AIPL1 also interacts with the NUB1 (NEDD8 ultimate buster 1), a protein that regulates proteasomal degradation. AIPL1 acts as a chaperone and functions to modulate NUB1 nuclear translocation [77]. The NUB1-binding site on AIPL1 is located between amino acid residues 181 and 330 in AIPL1 [72], with the C-terminal 144 residues displaying the highest ability of interaction [71]. This fact is to some extent in parallel with the finding that the sequence required for proper interaction of AIP with the AhR is within the most C-terminal 20 amino acids of AIP [78]. The retina-specific NUB1 protein isoform is ~16 kDa smaller than that in other tissues [71]. Unlike AIPL1, NUB1 is expressed broadly across diverse tissues of the eye, and it appears in the midperipheral and peripheral retinal regions earlier than AIPL1 during development [35]. Taken together with the known AIPL1-NUB1 interaction, this might indicate the role of AIPL1 in the regulation of the degradation of retinal proteins, thereby modulating cell signalling and cell growth at a certain stage of retina development. However, the interaction between AIPL1 and NUB1 is suggested to be transient during retinal development and only occurs as one of the AIPL1 functions [35,79].

AIPL1 was shown to cooperate with HSP70, but not HSP90, to suppress the formation of intracellular inclusions comprising misfolded fragments of NUB1 [31], which happens in a concentration-dependent manner [77]. Additionally, AIPL1 appears to regulate the stability of FAT10 (an ubiquitin-like modifier) itself and FAT10-conjugated proteins by opposing NUB1-mediated proteasomal degradation [73]. The effects of AIPL1 on NUB1-mediated degradation may occur through binding of AIPL1 to NUB1 and preventing NUB1 from productively associating with the proteasome to facilitate FAT10 degradation. Coexpression of AIPL1 with FAT10 leads to a ~30% increase in monomeric FAT10 and its conjugates, suggesting AIPL1 prevents FAT10ylated proteins from premature degradation [73]. AIPL1 also interacts directly with FAT10 [73], binding to both its N- and C-terminal ubiquitin-like domains, while ubiquitin was shown not to be the AIPL1 interactant, underlining a specific interaction with FAT10 [74]. The TPR motif plays a crucial role in binding FAT10, and proper folding of AIPL1 is necessary for FAT10 binding [74]. The residues of AIPL1 contributing to most of the interactions with NUB1 identified by computational modelling are Ser199, Lys243, Tyr247, Glu248, Lys265, Lys289, Glu292, and Glu294 [80]. Several pathogenic AIPL1 mutants, including Arg38Cys, Ala197Pro, Cys239Arg, and Pro376Ser, were shown to still interact with FAT10 [74], while Ala197Pro and Cys239Arg mutants fail to bind NUB1 [72], suggesting that AIPL1 might protect FAT10ylated substrates by sequestering NUB1. Not only does AIPL1 appear to block NUB1-mediated degradation of FAT10ylated proteins, but a small proportion of AIPL1 (~3.5%) itself is modified with FAT10 [73]. PDE6α and PDE6β were shown to be modified by covalent and non-covalent attachment of FAT10 and targeted for proteasomal degradation or down-regulated in the enzymatic activity of PDE6, respectively. In this process, AIPL1 forms a triple complex with PDE6β and FAT10 and slows down the degradation rate of monomeric FAT10 in a dose-dependent manner, resulting in an increased PDE6β-FAT10 conjugate formation. So it is quite possible that binding of NUB1 to AIPL1 is required to more effectively sequester FAT10 and keep it from inhibiting PDE6 or from mediating its degradation. It has been suggested that AIPL1 might capture FAT10 in the inner segment of photoreceptors, hinder its movement to the outer segment, and thus help to maintain the function of already exported PDE6 in the disc membranes [74].

### 3.3. PDE6 Maturation

AIPL1 is essential for the maturation of the PDE6 complex, a key enzyme in phototransduction [81]. The chaperone function of AIPL1 might ensure proper folding of PDE6 subunits and provide holoenzyme assembly before its trafficking to the outer segment [61]. In the absence of AIPL1, PDE6 subunits are rapidly degraded by the proteasome [75].

Interaction with PDE6 catalytic subunits mostly relies on the AIPL1 FKBP domain, especially on the insert region, as the AIPL1 Δ96–143 mutant was completely abolished in binding with the farnesyl probe [62]. AIPL1 interacts with PDE6α and PDE6β, with its association with PDE6β being dependent on PDE6α [75]. Farnesylation and geranylgeranylation of PDE6 catalytic subunits appear to influence their interaction with AIPL1. While AIPL1 preferentially binds farnesylated proteins [62,82], it also seems to interact with geranylgeranylated cone PDE6α′ [30,51]. It was hypothesized that AIPL1’s dynamic interactions with HSP90 and Pγ facilitate PDE6 maturation by promoting the proper conformation of the catalytic subunits [55], and later, the fact of the formation of a stable ternary complex between AIPL1, HSP90, and PDE6 subunits, which is crucial for PDE6 maturation, was proved [61]. Unlike some of the HSP90 clients, for example, Cdk4, GR, and AhR, PDE6 does not cycle between immature and functional states, and it matures irreversibly. Structural peculiarities of AIPL1-binding with HSP90 lead to a hypothesis that PDE6 is not threaded through the HSP90 dimer lumen like the above-mentioned proteins, but rather the highly dynamic nature of the AIPL1/HSP90 complex helps to induce the conformational changes in PDE6, leading to its maturation; in particular, conformational dynamics of AIPL1-FKBP may induce rearrangement of the catalytic domains in the PDE6 dimer [59].

Moreover, AIPL1 is implicated in protecting PDE6 from FAT10-mediated degradation. FAT10 was shown to covalently modify PDE6α, PDE6β, and PDE6γ, marking them for proteasomal degradation. However, AIPL1 can slow down this degradation by forming a ternary complex with FAT10 and PDE6β and stabilizing the PDE6-FAT10 conjugate [74].

Notably, AIPL1 appears to be localized predominantly from the synapse to the inner segment of PR cells in adults, with an enrichment in the connecting cilium [34], while the PDE6 complex is eventually trafficked to the outer segment [83]. This may suggest that AIPL1 chaperone function might be limited to PDE6 maturation and does not extend to the trafficking of PDE6 to the outer segments [84]. However, no univocal indication of AIPL1’s role in the relocation of PDE6 to the outer segment has been obtained to date.

## 4. *AIPL1* Dysfunction in Retinal Disorders

AIPL1 was initially discovered as a gene of the EST of the LCA4 candidate locus [19]. Although mutations in AIPL1 are mainly associated with autosomal recessive LCA and early-onset retinal dystrophy (EOSRD), AIPL1 is also linked to less severe IRDs of cone-rod dystrophy [28], juvenile or early-onset RP [28,85], non-early onset RP [86], and late-onset retinal degeneration [87]. From 2% [88] to 15% [89] of all LCA cases are caused by *AIPL1* mutations in different cohorts. Among the described pathogenic mutations in *AIPL1* [1], the following types are distinguished: point missense and nonsense substitutions and small indels (up to 18 bp), which might lead to splice variants that in turn alter exon composition, shift in reading frame with premature stop-codon, or in-frame indels [26,29]. Allele frequencies vary widely among the populations [90]; the most common pathogenic mutations appear to be p.Val71Phe, IVS2-2A>G, p.Gln141* [91], p.Gln163*, p.Trp278* [1]. In most cases, defects in both alleles of *AIPL1* are necessary for the manifestation of the disease, i.e., the inheritance pattern is autosomal recessive with homozygous or compound heterozygous alleles and a loss-of-function type of pathogenesis. Parents carrying one mutant allele generally do not experience any symptoms, but in some cases, mild visual impairments were described, such as significantly reduced rod function on ERG [40], probably due to haploinsufficiency. There were also reports about an adCORD and autosomal dominant juvenile RP caused by p.Ala352_Pro355del (also known as p.Pro351∆12bp) mutation in *AIPL1* [28]. On average, patients with homozygous p.Gly122Arg and compound heterozygous alleles, one of which is p.Gly122Arg, were shown to have milder and later-onset phenotypes like RP, amenable to therapeutic intervention [18,87,92,93]. Homozygous p.Trp278* usually causes the most severe symptoms with early onset [40,87,88,94,95].

*AIPL1*-caused LCA is at the severe and early-onset ends of the spectrum of LCA types [9,10]. Typical symptoms of LCA4 include a progressive loss of photoreceptors, markedly reduced or no response of photoreceptors to light on the ERG, less often keratoconus, cataract, nystagmus [28,40], in milder cases hyperopia, night blindness or light sensibility, poor vision, fundus with bone spicules pigmentation, variable degree of maculopathy, and optical coherence tomography (OCT) with reduced macular thickness [96]. Mostly, patients with LCA4 are congenitally blind or have severe visual impairments from the first few years of life, although cases of disease manifestation in more adult ages were also described [22].

### 4.1. Pathogenic Mechanism

Retinal phenotypes in mice with an *AIPL1* mutation were studied as an animal model for human *AIPL1*-caused retinal degeneration [63,81,97]. Analyses of *AIPL1* mutant mice revealed that AIPL1 is essential for the maintenance of both rod PDE6 α and β [75] and cone PDE6α′ subunits during development [67]. AIPL1 was shown to be required as an HSP90 co-chaperone for the stability of nascent PDE6 catalytic subunit polypeptides, their folding, proper assembly of the PDE6 complex, and its membrane association. Studies showed that in the absence of functional AIPL1, rod and cone PDE6 subunits are synthesized normally but rapidly degraded through the ubiquitin-proteasome system [67,75]. Since the PDE6 holoenzyme hydrolyzes cGMP, accumulation of cGMP in photoreceptors leads to their rapid degradation in the developing retina if *AIPL1* dysfunction takes place [63].

Although the role of AIPL1 as a co-chaperone of PDE6 is considered a generally accepted theory of pathogenesis, mouse models have shown that the reduction in PDE6 while *AIPL1* is functional affects the retina in a different way. In *Aipl1*-/- mice, both rod and cone photoreceptor cells degenerate at a similar rapid rate, whereas in retinal dystrophic mice, which result from a viral insertion and nonsense mutation (*rd1*) or missense mutation (*rd10*) in the rod *Pde6b* gene that causes a reduction in *Pde6b* mRNA and loss of rod PDE6 complex [98,99], rods degenerate faster than cones [63,99,100]. In *Aipl1*-/- mice, there is no recordable ERG at any age, whereas in both retinal dystrophic mouse models, there are some cone responses at postnatal day 12 [63,99]. This is consistent with the fact that in humans, mutations in *AIPL1* cause severe blindness that affects both rods and cones [87], whereas deficiencies in catalytic rod PDE6 subunits cause RP, primarily a rod disease [101,102], and deficiencies in cone PDE6α′ cause recessive cone dystrophy, and early-onset recessive complete and incomplete achromatopsia [103]. Overall, the functional deficits in the *AIPL1* mutant rods are more severe than those of the *rd1* mice. This heightened severity is likely because the loss of AIPL1 leads to the misassembly and proteasomal degradation of all PDE6 subunits, causing a catastrophic failure of the complex functionality. In contrast, the RP affects only one of the rod PDE6 subunits (PDE6β), which may allow for some residual activity of the complex or a partial loss of function. While this mechanistic distinction is sufficient to account for the phenotypic difference, it remains possible that AIPL1 is involved in other interactions, which, in the case of AIPL1 deficiency, could further aggravate the course of the disease [104].

The rod PDE6 α and β subunits were shown to be targets of FAT10ylation and a noncovalent interaction partner of this ubiquitin-like modifier FAT10 protein, leading either to proteasomal degradation of PDE6 or to a decreased PDE6 cGMP hydrolysis activity, respectively. AIPL1 stabilizes FAT10 and the PDE6β-FAT10 conjugate [74]. Also, AIPL1 appears to antagonize NUB1-mediated degradation of FAT10ylated proteins [73], while some pathogenic mutations of AIPL1 are defective in impeding this degradation [72,73], and mutations in NUB1 itself are not found to be associated with LCA [105]. It has been shown that FAT10 expression might be promoted by TNFα and IL-6 [106], and the production of these cytokines is triggered as a result of increased cGMP level caused by the inhibition of PDE6 with the drug Zaprinast in the porcine retinal explants [107]. This observation raises the hypothesis that FAT10 expression might be induced in the retina of LCA4 patients due to unproductive PDE6 assembly caused by pathogenic AIPL1 mutants. This in turn causes an inflammatory environment enabling FAT10 upregulation [74] that, in combination with impaired interaction of mutant AIPL1 with NUB1 [73], further enhances the FAT10-mediated degradation of PDE6 [74] and leads to subsequent rod degeneration. This theory is, however, inconsistent with the fact that only a few AIPL1 mutations impart the interaction with NUB1 and/or FAT10 [39,71,72,73,74,77].

Cone PDE6 is found predominantly in soluble retinal fractions, whereas rod PDE6 is found mostly membrane-associated, mediated primarily by the geranylgeranylated C-terminus of rod PDE6β [108]. Therefore, the mechanism of AIPL1 action during rod and cone PDE6 assembly and activity might diverge to accommodate these fundamental differences between rod and cone PDE6 [29].

The mechanism behind cone death in the case of loss of AIPL1 functionality seems to be different from that of rods and was believed to be the “bystander effect”, in which the viability of rods aids to support the survival of cones [42]. This phenomenon was first proposed to take place in RP [109], the disease that is caused by rod-specific photoreceptor-expressed genes and which starts from rod degeneration, ending up with the death of both photoreceptor types. However, in the absence of AIPL1 in cones, the subsequent disruption of RetGC1 (retinal guanylate cyclase-1) trafficking and its proteasomal degradation represents a critical secondary effect. RetGC1 mediates cGMP synthesis in response to changes in calcium level [67]. As a consequence of diminished levels of RetGC1, decreased cGMP levels lead to closure of cGMP-gated channels and are likely to reduce calcium levels below a threshold needed for long-term cone photoreceptor survival [67,110]. Although the mechanisms of the “bystander effect” have been revised recently, as discussed in detail in this review [111], it is likely that both these hypotheses (“bystander effect” and reduction in RetGC1) take place in cone photoreceptors; still, studies of cone death caused by *AIPL1* mutations are deficient.

It is believed that eventual photoreceptor cell death might happen due to excitotoxicity of Ca^2+^ accumulation [111,112], endoplasmic reticulum stress, oxidative stress, inflammatory responses, or their combination [111], most possibly by non-apoptotic cell death mechanisms [113]. However, it was not described explicitly for *AIPL1*-caused retina degradation in humans. cGMP-induced rod death in the case of PDE6 functional deficiency (rd1 mouse models) was proposed to be the following: elevated levels of cGMP activate CNG channels and/or PKG, causing excessive influx of Ca^2+^ and protein phosphorylation, respectively [113]. Then, possibly, PKG-dependent phosphorylation may trigger HDAC activation, which in turn could lead to PARP activation. Meanwhile, Ca^2+^ influx might independently or simultaneously activate calpains [114]. Both pathways act synchronously to cause photoreceptor cell death, but surprisingly, this cGMP-induced form of cell death appears to occur 4–6 times slower than apoptosis and shares certain features with PARthanatos. Interventional experiments in the rd1 mouse confirmed the presence of this pathway and the connections between the different metabolic processes by demonstrating the neuroprotective effects of inhibition of PKG, calpain, PARP, and HDAC [113]. Cone cell death was shown to be nonautonomous necrosis dependent on Rip3 kinase in the Pde6β-deficient mouse model (*rd10*) [115]. Understanding the particular mechanisms of photoreceptor cell death and knowledge about potential targets for inhibition of this process are crucial for extension of the therapeutic window.

### 4.2. AIPL1 Mutations Identified in the IRDs

Speaking about specific mutations in *AIPL1*, described as patient cases, some of them were investigated in vitro on structural stability, intracellular localization, interaction with HSP90, PDE6 subunits, NUB1, and FAT10. Studies have shown that most of the tested mutant variants retain normal general structure and localization, while some of them undergo misfolding, aggregate, and form inclusion bodies or become mislocalized. This has been shown, for example, for p.Arg53Trp [18], p.Gly64Arg, p.Trp72* [29], p.Trp72Ser [51], p.Trp88* [29] p.Cys89Arg [30], p.His93Ala-fs*66, p.Glu215Ala-fs*3 [29], p.Trp278* [29,77,116], p.Glu282_283dup [29] p.Ala336del2 [116] pathogenic variants of AIPL1, and most of them are impaired in protein–protein interaction ability as a consequence. Two possible mechanisms of their pathogenicity were suggested: aggresomes of mutant AIPL1 accumulate in the photoreceptors of LCA patients carrying such mutations, and this mislocalization and accumulation trigger apoptosis, or alternatively, the pathology may simply arise from an absence or insufficiency of functional AIPL1 protein [116]. The next group of mutations that do not significantly impair global protein conformation but reduce interaction of AIPL1 with HSP90 and/or impair its ability to modulate PDE6 activity includes p.Leu17Pro [29], p.Thr39Asn [18], p.Gly64Arg [29] p.Trp72Arg [18,51], p.Cys89Arg [29,51], p.Cys89Tyr [18], p.Gln163* [29], p.Tyr194*, p.Ser198Phe, p.Trp222* [18], p.Cys239Arg [31], p.Glu245*, p.Arg270His [18], p.Glu282_283dup [29] p.Leu293Pro, p.Glu309Asp-insLNRREL [18]. Some of the variants, like p.Val71Phe [29], p.Gly122Arg, p.His130Gln [18] has been shown to retain normal ability to bind to HSP90, while interaction with PDE6 was impaired; notably, p.Gly122Arg and p.His130Gln are proposed hypomorphic variants associated with milder later-onset retinal dystrophy [18]. Also, AIPL1 mutations p.Val96Ile [71], p.Ala197Pro, p.Cys239Arg [72,73] failed to interact with NUB1, and the last two were impaired in their ability to antagonize NUB1-mediated degradation of FAT10 and FAT10 substrate [73].

Two specific p.Pro366Arg and p.Pro376Ser variants achieve enhanced modulation of PDE6 activity precisely through a reduced interaction with HSP90 [18], although their association with retinal dystrophy was questioned recently, which is discussed further. It is worth noting that the role of HSP90 in retinal diseases is equivocal: on the one hand, inhibition protects against inherited retinal degeneration [117]; on the other hand, prolonged HSP90 inhibition leads to degradation of its clients and ocular toxicity in the retina [57]. This suggests the outcome of perturbing the AIPL1-HSP90 interactions is highly dependent on the molecular context, and the precise regulation of the AIPL1-HSP90-PDE6 axis is critical for normal photoreception.

There were disagreements about the association of *AIPL1* PRD mutations with retinopathies. Most of them were shown not to alter the secondary structure, stability, and interaction of AIPL1 with HSP90 and PDE6α [52], which raised the uncertainties about their pathogenicity. Missense p.Arg302Leu substitution initially was described as a mutation that occurs in LCA patients [28], but its pathogenic role was disproved by finding biallelic disease-causing mutations in other genes in patients [88,89] and homozygous p.Arg302Leu variants in an unaffected person [88]; however, recent multilateral computational analysis revealed a possible digenic mechanism of mutations in *AIPL1* and BBS2 to contribute to the retinal dystrophy development [118], but no experimental validation was obtained. p.Pro376Ser was also reported as an LCA mutation [28], but its pathogenic role was questioned repeatedly due to its relatively high frequency in the control cohort, in silico analysis tools’ predictions, and in vitro tests [18,72,88]. The situation with the p.Ala352_Pro355del in-frame deletion is even more breathtaking: it was stated as the first known monoallelic disease-causing *AIPL1* mutation in CORD and jRP patients [28]; then it was studied in mouse models, which confirmed the dominant effect of this mutation and the inability of AIPL1 with this deletion to support a normal level of PDE6 subunits [66]; however, structural analysis and in vitro binding assays did not reveal any significant difference between this variant and wild-type *AIPL1* [52], except for a decreased thermal stability [116]. The mechanism of gain-of-function pathogenicity caused by this mutation was proposed to be one of the following. p.Ala352_Pro355del interferes with PDE6 folding or assembly independently of wild-type AIPL1. As AIPL1 was identified to be a partner of the Pγ subunit [52,55], this interaction might also be altered by the p.Ala352_Pro355del mutation, although AIPL1-Pγ interaction was shown to be independent of PRD, and the PRD mutations did not appreciably affect the affinity of hAIPL1 for Pγ [52]. Or the pathogenic effect of p.Ala352_Pro355del might be mediated via as yet unidentified photoreceptor-specific interacting proteins. Still, the patient’s case reports about PRD variants are in deficit, and these and other *AIPL1* mutations require more detailed investigation.

Alternative splice variants, like in-frame skipping of exon 3, were detected as minor transcripts from wild-type *AIPL1* in the in vitro splice assay, and a low abundance of this isoform was also detected in vivo in normal retina [24,26]. Case studies show that mutations associated with retinal degeneration of a wide spectrum of severity occur in the exon 3 [18,28,40,41,85,86,88,91,119,120,121,122,123,124,125,126] and in vitro assays support the indispensability of this region for *AIPL1* function [26,61,62,71,116,127]. Some mutations were shown to induce skipping of exons in AIPL1 transcripts and, therefore, might shift the relative abundance of splice isoforms in the retina. Among them, c.465G>T, which has been shown to be a splice mutation leading to exon 3 skipping [26] and identified as a homozygous mutation in all affected members of a consanguineous family diagnosed with retinal degeneration, with heterozygous carriers being unaffected [126]. These findings suggest that there is a tolerance threshold for the expression of this naturally occurring *AIPL1* splice variant [26]. Other mutations affecting *AIPL1* splicing were reported to occur in patients: c.276+1G>A, c.276+2T>C [128] (both lead to in-frame exon 2 skipping, exons 2 and 3 skipping, or frame-shift, occur as compound heterozygous) [26] and c.642G>C [129] (previously thought to be a missense substitution p.Gly262Ser, in vivo leads to in-frame exon 4 skipping or in-frame insertion of 7 amino acids, occurs as a compound heterozygote) [26], and this confirms the importance of the presence of a high proportion of full-length transcripts. There was a revision of *AIPL1* variants c.465G>T, c.642G>C, and c.784G>A, which were previously considered to be missense mutations p.Gln155His, p.Lys214Asn and p.Gly262Ser, respectively, but the in vitro splicing assay revealed that they alter transcripts processing yielding aberrant splice products which disrupt the domain organization of the AIPL1 protein [26].

Undetected variants in the promoter, untranslated regions, cis-acting or regulatory elements within the introns of *AIPL1*, such as splicing enhancers and silencers, may influence gene expression, RNA stability, or splicing, thus contributing to disease. It implies that the prevalence of *AIPL1*-associated LCA might be underestimated [26,130]. Also, nothing is known so far about epigenetic and siRNA regulation of AIPL1 expression, RNA editing, and other molecular mechanisms of modulation of *AIPL1* expression and functioning. Moreover, the mechanism of pathogenicity of missense and in-frame indel mutations might include the alteration of post-translational modification of certain amino acid residues of AIPL1, which is complicated to study in the in vitro expression systems.

The detailed information about published pathogenic and likely mutations in *AIPL1* described above and many others [1,9,12,18,22,28,29,39,40,41,86,87,88,89,90,91,92,93,94,119,120,121,122,123,124,125,126,127,128,129,131,132,133,134,135,136,137,138,139,140,141,142,143,144,145,146,147,148,149,150,151,152,153,154,155] has been compiled into Table 2 and designated with dots on Figure 1. It is worth mentioning that the presented table provides a large number of monoallelic heterozygous cases that are considered to be associated with LCA or another retinopathy, while only one *AIPL1* variant, p.Ala352_Pro355del, is shown to cause autosomal dominant CORD and jPR [28,66]. The detection of heterozygous variants in autosomal recessive loss-of-function disorders may be explained by several possibilities. A deletion or duplication on the other allele could go unnoticed if copy number variant analysis were not conducted. Additionally, screening might not cover deep intronic regions, so variants located there would remain undetected [124,156,157,158]. Moreover, as very little is known about *AIPL1* regulatory elements, their variants located distantly from the gene might not be ruled out [147]. Finally, the causative mutations could have been located in another gene that was not investigated in the particular research.

The diagnosis of LCA is made through a combination of clinical, electrophysiological, and molecular genetic approaches. Clinically, affected infants present within the first months of life with severe visual impairment and absent or sluggish pupillary responses. Fundus changes may be mild in early infancy but can progress to show pigmentary retinopathy or atrophic changes resembling severe RP. A hallmark finding is the absence or severe reduction in electroretinographic (ERG) responses, which reflects global dysfunction of rod and cone photoreceptors [40]. The definitive diagnosis relies on genetic testing, most commonly genotyping microarray for inherited retinal dystrophies or whole-exome sequencing. The LCA genotyping microarray had been considered as a robust and cost-effective first-line screening tool for this genetically heterogeneous disorder. By enabling simultaneous interrogation of all known LCA-associated variants in large patient cohorts, it allows us to systematically identify pathogenic alleles, thereby supporting early molecular diagnosis and disease progression prognosis [124,142,145]. Importantly, this approach was broadly applicable, as it does not rely on detailed clinical phenotyping for initial decision-making and can efficiently guide subsequent targeted sequencing [159]. However, it is now considered that the LCA microarray might be used only as the first step in the molecular diagnosis, as the likely causative gene can be identified in approximately a third of patients [142]. Newer techniques, such as next-generation sequencing (NGS), enable a higher rate of mutation detection. As the cost decreases, NGS is used more commonly [88,160]. This technology has proven robust for identifying both novel and known mutations in candidate gene panels by targeted capture [119,121] or by exome or whole genome sequencing [119,161]. Currently, NGS has become the method of choice in determining the causative mutations in IRD patients [160]. The early diagnosis of LCA is essential, as it opens up a possibility for effective gene therapy [162], which will be discussed further.

## 5. Model Systems

The research of LCA caused by *AIPL1* mutations relies on a multi-faceted approach that uses various in vitro and in vivo models. Each of these model systems offers unique advantages and has its own limitations, facilitating the investigation of disease mechanisms and the development of therapies. Cell models provide a controlled, modifiable, and accessible system for initial mechanistic studies and high-throughput screening of therapeutic candidates, while animal models serve as essential preclinical surrogates, providing the complex living systems necessary to evaluate a therapy’s efficacy, safety, and systemic biological response before clinical trials.

Human retinoblastoma-derived cell lines, such as Y79 and Weri-Rb1 [34,71,74], have been used as a model for photoreceptor-like cells due to their neuronal origin and expression of photoreceptor-specific markers. These cell lines have been invaluable for elucidating the fundamental biology of the AIPL1 protein. High endogenous levels of AIPL1 in retinoblastomas, as confirmed by later studies [33], make them a relevant in vitro system for investigating protein–protein interactions and cellular localization. In particular, the interactions between AIPL1 and its partner protein NUB1 were first identified in Y79 cells [71], whereas interaction with FAT10 and its functional background was described in Weri-Rb1 [74]. The finding that the level of *AIPL1* expression in retinoblastoma cells is comparatively high [33] validates the use of these cancer cell lines for studies of AIPL1 interaction properties.

The ARPE-19 cell line, spontaneously arisen from human retinal pigment epithelium (RPE) [163], has been used as an in vitro model [164] for testing therapeutic strategies. It is well-established that with increasing passage number, ARPE-19 cells can de-differentiate, lose their morphology, pigmentation, and protein markers related to cell function. The effects of culture media and extracellular matrix coatings on the ARPE-19 differentiation state were described [165,166]. Treatment of ARPE-19 by the synthetic retinoic acid derivative, N(4-hydroxyphenyl) retinamide, also known as fenretinide, induced a neuronal-like phenotype in the human adult retinal pigment epithelial cell line [167,168]. Specifically, the ARPE-19 cell line has been utilized in early gene therapy vector development, for evaluating the in vitro transduction efficiency of different AAV serotypes [169]. While ARPE-19 cells have provided valuable insights, it is important to note their limitations in modelling photoreceptor-specific processes, as they originate from the RPE rather than photoreceptors. Nevertheless, their ease of culture, trans-differentiation with fenretinide, and susceptibility to viral transduction make them a useful preliminary screening tool for evaluating *AIPL1* expression constructs and viral vectors before proceeding to more complex models.

Introduction of genes of two transcription factors, *CRX* and *NEUROD1*, to the human iPSCs was shown to generate photoreceptor-like cells. The resulting cells expressed phototransduction-associated genes and exhibited functional properties in calcium imaging; however, they do not elaborate a complete photoreceptor cell ultrastructure, including connecting cilia and outer segments. Nonetheless, this simple one-step approach might be used for IRD modelling and drug discovery [170]. For example, it is possible to produce *AIPL1* knockout iPSCs with the help of the CRISPR/Cas9 editing system to model LCA4 photoreceptors in vitro, as was completed on another model system in another work [171], which is discussed below.

A more pertinent option for in vitro modelling is retinal organoids. First, LCA4 modelling organoids were derived from iPSCs, which were reprogrammed from cells of LCA patients harbouring a biallelic *AIPL1* mutation. These organoids retained a well-developed outer nuclear layer (ONL) with rod and cone photoreceptors displaying a substantial reduction in AIPL1, PDE6α, and PDE6β protein and an increase in cGMP levels [138,144], thus being a relevant resource for LCA4 therapeutic testing. Despite this undeniable advantage, patient-derived cells are subject to ethical considerations, and the cost-prohibitive scalability might limit their industrial and clinical applications. To address this restriction, isogenic retinal organoids were formed from *AIPL1* CRISPR/Cas9 knockout iPSCs generated from fibroblasts. These retinal organoids effectively recapitulate key molecular characteristics of LCA4, demonstrating alignment with previous in vitro models derived from patient cells and supporting the model’s validity as a screening tool [171].

Zebrafish models of IRDs are considered a suitable animal model system to study IRDs in a cone-rich retina and to check potential therapeutics. Zebrafish are also known to regenerate their retina after injury through the reprogramming of Müller glia, thereby providing a valuable model for testing strategies that exploit this natural regenerative ability [172]. The *gold rush* (*gosh*) zebrafish model, which spawned from a mutation in the cone-specific *aipl1b* gene, has been described. Cone photoreceptors of this model undergo progressive degeneration, and Pde6α′ is markedly decreased or absent, while its mRNA level is not affected [16]. This is consistent with the known function of AIPL1 in human photoreceptors [67], making *gosh* zebrafish a convenient model to study AIPL1 function in cones. Yet, while zebrafish retained two *AIPL1* genes acting in rod and cone photoreceptors, respectively, humans have only one *AIPL1* [15], which is expressed both in rods and cones during retina development, and in the adult human retina, its expression is higher in rods [35,38,39]; also, *gosh* zebrafish demonstrate primary cone degeneration [16] as opposed to the human LCA4 phenotype, which mostly starts from the death of rods [39]. Additionally, human Müller glial cells have very limited reprogramming potential, unlike those in zebrafish [173,174]. Thus, *gosh* zebrafish are not completely modelling the human LCA4 phenotype, and more fitting animal models are required for LCA4 therapy testing.

Retinal degeneration caused by *AIPL1* defects has been described in several mouse models of LCA4 [63,81,97]. In *Aipl1*-deficient (“null”) mouse models, photoreceptor outer segments were shorter than normal and disorganized by postnatal day 11 [63]. Retinal degeneration was first detected at postnatal day 12 [63,97], leaving only a single layer of photoreceptor nuclei remaining at postnatal day 18. Complete photoreceptor degeneration was observed after four weeks in one model [63], and most of the photoreceptors were lost by eight weeks in another model [97]. A different knockdown approach was used to produce a hypomorphic mutant in which *AIPL1* expression was reduced, but not eliminated. In this mouse model of LCA, the rate of degeneration was slower, and the thickness of the photoreceptor layer was normal at three months of age, despite disorganized outer segments of photoreceptors. By eight months of age, over half of the photoreceptors were lost, and the photoreceptor outer and inner segments were shortened [81]. In all these mouse models of LCA4, the overall morphological development of the rod and cone photoreceptors appeared to be normal. However, reduced expression of AIPL1 in mice resulted in a delay in photoresponse onset and recovery prior to retinal degeneration [81], whereas in the complete absence of AIPL1, a recordable ERG could not be detected at any age [63,97]. This parallels the human condition, as LCA patients with mutations in *AIPL1* typically lack a recordable ERG within the first year of birth. This indicates that although the photoreceptors may appear morphologically normal during development, AIPL1 is essential for the normal functional development of the photoreceptors. It is therefore plausible that in this LCA patient’s retina, both rod and cone photoreceptors, including the photoreceptor inner and outer segments, underwent normal morphological development, but remained non-functional, and that photoreceptor degeneration proceeded after birth [39]. These mouse models (null and hypomorphic) represent two ends of the LCA4-RP spectrum associated with *AIPL1* defects in humans.

A mouse model of autosomal dominant CORD caused by the p.Ala352_Pro355del mutation in *AIPL1* was also created [66]. This model played a significant role in unravelling the molecular mechanism of this disease and highlighted a promising therapeutic avenue for patients with adCORD. Mice expressing the mutant human AIPL1 (p.Ala352_Pro355del) developed early and significant defects in cone-mediated vision, followed by progressive degeneration of photoreceptors, closely resembling human CORD. The disease phenotype was dominant: even when both mutant and normal *hAIPL1* were present, vision defects persisted, indicating the mutation’s strong negative effect on retinal function. The dominant effect of the p.Ala352_Pro355del mutation was observed in both cones and rods, with mutant mice showing significantly reduced ERG responses compared to controls [66]. Although murine AIPL1 lacks PRD [14] and the retina of the mouse differs significantly from that of humans [175], the discussed mouse models successfully recapitulate the LCA4 and adCORD phenotype and were used for testing of AAV-based gene therapy, thus proving their applicability in drug testing [48,66,176].

For preclinical testing, one of the most suitable models is a feline in vivo model of AIPL1-related retinal dystrophy that has been identified and genetically characterized [17]. This model is a population of Persian cats that suffer from an inherited form of LCA due to a nonsense mutation in the *AIPL1* gene. These cats carry a c.577C>T, resulting in a generation of early stop codon p.Arg193* in AIPL1, which leads to production of a dysfunctional truncated protein. This mutation causes early and severe progressive photoreceptor loss, with retinal degeneration occurring prior to adulthood and leading to blindness, making these cats a useful model of *AIPL1*-associated LCA [17].

Each model is purposefully selected to answer specific biological and therapeutic questions, as no single preclinical model is sufficient on its own. The journey from discovery to therapeutic application begins with accessible, high-throughput in vitro systems ranging from easy-to-cultivate retinoblastoma cell lines and versatile ARPE-19 cells to physiologically relevant retinal organoids. These models provide the initial testing ground for elucidating molecular mechanisms and conducting preliminary vector screening. This work then culminates in the comprehensive preclinical assessment enabled by a suite of in vivo animal models: zebrafish offer insights into cone-specific degeneration and regeneration, mouse models provide a granular view of the rapid degenerative cascade and functional deficits, and the feline model represents a critical large-animal surrogate for evaluating therapeutic efficacy and delivery in a human-sized eye.

## 6. AAV-Mediated Gene Therapy in AIPL1-Caused Retinal Disorders

The reported survival of cone-like photoreceptors in the retinas of some LCA4 patients at over 20 years of age is encouraging. Although these cone-like cells are completely non-functional and lack any photoreceptor ultrastructure, this might allow for the possibility of therapy at a younger age to rescue the surviving photoreceptor cells [39]. There was also promising evidence that in the very youngest *AIPL1* LCA patients, there is relative preservation of foveal outer retinal structure, even with the most commonly observed severe sequence variant [128]. These results suggest that therapeutic intervention is possible and appropriate in cases of late-onset, slower forms of *AIPL1* disease, like RP [87], or must otherwise be initiated in the first few years of life [94], as the window for therapeutic intervention is narrow in severe LCA4 cases. It should also be kept in mind that retinal degeneration affects not only the photoreceptor cell layer but also other types of retinal neurons, which also undergo irreversible changes [177], and the choice of an appropriate therapy strategy should be provided in each case.

While the possibility of various treatment options for IRDs was described previously (for example, translational readthrough-inducing drug [144], photoreceptor transplantation [178], and gene editing [13]), in this review, we will focus only on the already existing AAV-based vectors for *AIPL1*-associated RD. Carrying vector choice, methods of addressable therapeutic delivery, and challenges of gene therapy for ophthalmological conditions are reviewed in a recent paper [179]. Situations when gene augmentation therapy is not helpful already and when retina transplantation is more suitable are nicely discussed in another review [180].

As a unique and highly specialized extension of the central nervous system, the eye offers several distinct advantages for gene augmentation therapy. It is easily accessible and contains a natural subretinal space, where a bolus of therapeutic solution can be delivered relatively safely and without leakage into the systemic circulation [181]. Also, the eye has immune privilege, meaning that it is able to suppress immune responses to protect vision from inflammation [182]. Finally, visual function and retinal structure can be routinely and easily assessed after injections with noninvasive advanced technologies, such as visual acuity, contrast sensitivity, fundus autofluorescence, dark-adapted visual threshold measurement, vascular diameter analysis, pupillometry, ERG, multifocal ERG, and OCT [181]. Given these unique features of the eye, IRDs are one of the most attractive targets for gene therapy [162]. Approval of the first gene therapy to treat an IRD—voretigene neparvovec-rzyl (Luxturna)—a recombinant AAV expressing the RPE65 gene for the treatment of LCA type 2 [183]—opened up the potential for gene therapy of other IRDs. AAV is the most successful gene delivery vector and the standard choice for transduction of photoreceptors [184]. Naturally occurring and engineered AAV serotypes have been shown to efficiently transduce in vitro models [169] and photoreceptors in animals [185,186]. Due to its small size (~1.2 kb), the *AIPL1* CDS [22] can be efficiently packaged in AAV, allowing free choice of regulatory elements [187,188].

*AIPL1*-caused RDs are predominantly inherited in an autosomal recessive manner and thus are susceptible to gene supplementation therapy with AAV delivery of a healthy gene variant. However, the mouse model evinced that autosomal dominant CORD and RP caused by monoallelic *AIPL1* p.Ala352_Pro355del mutation are also amenable to treatment by gene augmentation [66]. AAV-mediated overexpression of wild-type *hAIPL1* in mutant p.Ala352_Pro355del hAIPL1 mice rescued cone-mediated vision with drastic improvement of photopic ERG responses and restoration of cone transducin (Gat2) and PDEa′ expression, deficient in mutant mice prior to degeneration, although recovery of rod photoreceptor function could not be achieved even at 50-fold AIPL1 overexpression. The therapeutic effect was stable for at least six months post-treatment, demonstrating the potential of AAV-mediated gene therapy for *AIPL1*-related adCORD [66].

AAV preparations of 2 and 8 serotypes carrying human and mouse *AIPL1* gene sequences were tested on hypomorphic [81] and null [63] *AIPL1* deficiency mouse models. Therapy preserved photoreceptor cells and maintained retinal function in both slow and rapid degeneration models. AAV2/2 vectors were effective in the slower-degenerating model, while AAV2/8 vectors worked in both deficiency models, demonstrating flexibility and versatility in addressing different disease severities. This research provides evidence of long-term (over 1 year) rescue of photoreceptor-specific defects using gene replacement, even in rapidly degenerating retinas. The findings support the potential for gene therapy to treat the broad clinical spectrum of LCA4, from milder to more severe phenotypes [48].

In recent research, trans-differentiated ARPE-19 cell lines with fenretinide have been transduced with AAV vectors carrying either wild-type or codon-optimized *AIPL1* gene variants to evaluate transduction efficiency and transgene expression. RNA-seq analysis of trans-differentiated ARPE-19 cells transduced with AAV9-*AIPL1co* (codon-optimized) demonstrated a differential decrease in expression of genes involved in the innate immune response, with AAV9-*AIPL1wt* (wild-type) vector inducing prominent activation of interferon-stimulated genes. This study revealed that cells transduced with AAV9-*AIPL1co* exhibited significantly less antiviral response compared to those transduced with wild-type *AIPL1*, suggesting that nucleic acid sequence, in particular codon optimization, may affect immunogenicity, which is a crucial consideration for gene therapy applications [189]. Although AIPL1 is not endogenously expressed in the RPE [34], the pigment epithelium serves as a primary innate immune-competent barrier in the retina. From this point of view, the use of ARPE-19 cells provided a somewhat controlled environment to evaluate the impact of codon optimization on innate immune activation. The reduced immune response seen with the optimized variant highlights the potential of this model for screening the immunogenic profiles of gene therapy constructs. Therefore, a crucial unanswered question is whether codon-optimized AIPL1 would offer the same immunogenic benefits when delivered specifically to its native cell type. To address this, future studies in more relevant models, such as retinal organoids, will be essential to determine if the reduced immunogenicity observed in vitro translates to photoreceptor cells where AIPL1 is naturally expressed.

The next study tested a novel gene therapy using a self-complementary Y733F mutant AAV2/8 capsid (sc-Y733F-AAV), which was designed for faster and higher gene expression—a determinative characteristic of a vector for rapidly degenerating retinal tissues. Treatment with sc-Y733F-AAV led to significantly greater preservation of photoreceptors and functional vision in *AIPL1* null mice [63] compared to the single-stranded vector AAV2/8 (ssAAV8). Notably, only the sc-Y733F-AAV vector achieved vision rescue when administered during the active phase of retinal degeneration, highlighting its potential for treating aggressive, fast-progressing forms of LCA [176].

Gene therapy vector rAAV2.7m8.hRK.*AIPL1* was tested in two human retinal organoid models of LCA4 [144,171]. It successfully restored key molecular features of healthy photoreceptors, including rescuing the loss of rod PDE6 and reducing abnormally high levels of cGMP, both hallmarks of AIPL1 deficiency. The results in organoid models provided strong preclinical support for moving toward clinical trials in patients with *AIPL1*-associated LCA [190].

Approaching the culmination of the story about gene therapy of *AIPL1*-associated retinal degeneration, one cannot help but mention an open-label, first-in-human interventional study, the results of which were published recently and which directly showed that intervention by gene supplementation therapy was safe and could improve outcomes in children with LCA4. This clinical study involved four children aged 1–3 years with severe retinal dystrophy associated with biallelic pathogenic sequence variants in *AIPL1*. Prior to treatment, their binocular visual acuity was limited to light perception. A recombinant adeno-associated viral vector containing the human *AIPL1* CDS under control of the human rhodopsin kinase promoter region (rAAV8.hRKp.*AIPL1*) was developed and administered to one eye of each patient by subretinal injection. At an average follow-up of 3.5 years (range 3.0–4.1), the treated eyes showed significant improvement in visual acuity, whereas the untreated eyes had deteriorated to the point of being unmeasurable. In the two children able to perform objective visual acuity tests, improvements in visual function were confirmed, and visual evoked potential recordings demonstrated increased visual cortex activity specific to the treated eyes. In three of the children, the outer retina was better preserved in the treated eye than in the untreated eye, and retinal thickness appeared better maintained in the treated eye for all four participants. The treated eye of only one child developed cystoid macular edema, and no other safety concerns were observed [12]. This study is indeed a major milestone in the progress of treatment for *AIPL1*-related LCA. This therapy was manufactured and provided under a UK Medicines and Healthcare Products Regulatory Agency (MHRA) Specials Licence, allowing for the use of unlicensed medicines to meet the special needs of individual patients, when no licensed alternative is available. The MHRA has also granted the therapy an Innovative Passport Designation, which can accelerate time to market and patient access [191]. Currently, the developer MeiraGTx seeks expedited approval [192].

The summary of the existing AAV-vectors developed against AIPL1-related retinal degenerations is provided in Table 3.

## 7. Conclusions

Unique anatomical and immunological properties of the eye make it an ideal target for gene therapy. Its small size, compartmentalization, and immune privilege enable precise viral vector delivery to specific retinal cell types, minimizing systemic dissemination and immune reactions. The optical transparency of the eye makes it highly accessible and facilitates non-invasive techniques to monitor and measure the effects of treatment. As such, diseases of the eye are prime candidates for gene therapy approaches.

Understanding the molecular mechanisms of AIPL1 functions and the pathogenesis of *AIPL1*-associated LCA creates the bridge between basic science and clinical translation. This review has detailed the unique structural biology of the human AIPL1 protein, revealing how its specialized domains act as a critical co-chaperone for the phototransduction enzyme complex PDE6 and possibly other retinal proteins. The elucidation of its pathogenic mutations provides a clear mechanistic basis for the severe retinal degeneration observed in LCA4, characterized by the rapid loss of both rod and cone photoreceptors due to failed PDE6 maturation and toxic cGMP accumulation.

All the preclinical data have been obtained by methodological research and refined through a purpose-built hierarchy of models. Usage of high-throughput in vitro systems and physiologically relevant in vivo models has played an indispensable role in de-risking therapeutic candidates.

The efficiency of AAV-mediated gene delivery was demonstrated by the recent first-in-human trial of AAV8.hRKp.*AIPL1*, which enabled successful transduction and led to significant improvements in visual function in children, marking a historic milestone for LCA4. This achievement validates decades of research and underscores that the window for intervention, though narrow, is clinically accessible.

Looking forward, the path is set for the next generation of therapies. The lessons learned from codon optimization to reduce immunogenicity, the refinement of AAV serotypes and promoters, and the emerging potential of gene editing and other modalities promise to enhance efficacy and expand the target patient population. We hope that the story of *AIPL1* is no longer one of irreversible blindness but one of pioneering science successfully bridging the gap between a genetic diagnosis and a transformative therapeutic reality, offering a tangible template for tackling other IRDs.

## Figures and Tables

**Table 1:** AIPL1 interacting partners.

Interactant	Method	Source
HSP70	yeast two-hybrid system, in vitro biochemical assays	[31]
HSP90	yeast two-hybrid system, in vitro biochemical assays	[31]
	immunoprecipitation, quantitative ELISA	[29]
	bio-layer interferometry, cross-linking/SDS-PAGE, cross-linking/mass photometry	[61]
NUB1	yeast two-hybrid system, co-immunoprecipitation/immunoblot, immunohistochemistry	[71]
	immunohistochemistry, subcellular fractionation/immunoblot	[35]
	yeast two-hybrid system	[31]
	yeast two-hybrid analysis, immunoprecipitation/immunoblot, GST pull-down/immunoblot	[72]
FAT10	immunocytochemistry, immunoprecipitation/immunoblot, GST pull-down/immunoblot	[73]
	immunoprecipitation, in vitro biochemical assays	[74]
rod PDE6α (*PDE6A*)	immunoprecipitation/immunoblot, mass spectrometry	[75]
rod PDE6β (*PDE6B*)	immunoprecipitation/mass spectrometry	[75]
cone PDE6α′ (*PDE6C*)	retinal extracts, immunoblotting, immunoprecipitation/immunoblotting	[67]
rod Pγ (*PDE6G*)	fluorescence binding assay	[52]
	dynamic light scattering, biolayer interferometry, fluorescence binding assays, NMR spectroscopy	[55]

**Table 2:** Cases of AIPL1-associated retinal dystrophy.

Inheritance Pattern	Allele 1			Allele 2			Cohort	Conditions	Reference
	cDNA	Protein	Type	cDNA	Protein	Type			
n/s	c.26-delT	p.Val9-del1	fs	n/s	n/s	n/s	n/s	LCA	[1]
n/s	[n/s] insGGAA	p.Val9-ins4	fs	n/s	n/s	n/s	n/s	LCA	[1]
compHet	c.34dup	p.Val12Gly-fs*32	fs	c.238C>T	p.Arg80Trp	mis	German	LCA	[131]
hom	c.40A>G	p.Lys14Glu	mis	c.40A>G	p.Lys14Glu	mis	Indonesian	LCA	[132]
compHet	n/s	p.Leu17Pro	mis	n/s	p.Lys214Asn	splVar	Vietnamese	LCA	[94]
compHet	c.59delG	p.Gly20Ala-fs*14	fs	c.421C>T	p.Gln141*	nons	Chinese	LCA	[91]
compHet	c.94C>T	p.Arg32*	nons	c.276+2T>C	[IVS2+2T>C]	splVar	n/s	LCA	[128]
compHet	n/s	p.Val33-fs	fs	[c.834G>A]	p.Trp278*	nons	European	LCA	[87]
n/s	c.97insGTGATCTT	p.Val33ins8	fs	n/s	n/s	n/s	n/s	LCA	[1]
compHet	n/s	p.Val33ins8bp	fs	[c.834G>A]	p.Trp278*	nons	U.S. American	LCA	[40]
compHet	c.97_104dup or c.96_97 insGTGATCTT	p.Gly31fs [p.Phe35-fs]	fs	c.IVS5-10_786del (c.785-10del12CTCCCCACAGGC)	[IVS5-10del12bp]	splVar	Italian	LCA	[93]
compHet	c.96_97insGTGATCTT	p.Phe35Leu-fs*2	fs	c.785-10del12CTCCCCACAGGC	c.IVS5-10_786del [IVS5-10del12bp]	splVar	n/s	LCA	[128]
compHet	c.96_97insGTGATCTT	p.Phe35Leu-fs*2	fs	c.834G>A	p.Trp278*	nons	n/s	LCA	[128]
compHet	c.98_99 insTGATCTTG	p.Phe35-fs*36	fs	c.834G>A	p.Trp278*	nons	Italian	LCA	[93]
compHet	c.98_99insTGATCTTG	p.Ile34Asp-fs*10	fs	c.834G>A	p.Trp278*	nons	n/s	LCA	[128]
het	c.111delC	p.Arg38-fs	fs	-	-	-	Spanish	LCA, non-early-onset RP	[86]
n/s	c.112C>T	p.Arg38Cys	mis	n/s	n/s	n/s	n/s	LCA	[1]
hom	c.116C>A	p.Thr39Asn	mis	c.116C>A	p.Thr39Asn	mis	Pakistani	LCA	[133]
							n/s	LCA	[18]
compHet	n/s	p.Cys42*	nons	[c.784G>A]	p.Gly262Ser		U.S. American	LCA	[40]
hom	c.152A>G	p.Asp51Gly	mis	c.152A>G	p.Asp51Gly	mis	Chinese	EOSRD	[91]
compHet	c.152A>G	p.Asp51Gly	mis	c.182delT	p.Ile61Thr-fs*43	fs	Chinese	EOSRD	[91]
compHet	c.152A>G	p.Asp51Gly	mis	c.325C>T	p.Gln109*	nons	Chinese	EOSRD	[91]
compHet	c.152A>G	p.Asp51Gly	mis	c.421C>T	p.Gln141*	nons	Chinese	EOSRD	[91]
compHet	c.152A>G	p.Asp51Gly	mis	c.506 T>C	p.Leu169Pro	mis	Chinese	EOSRD	[91]
compHet	c.152A>G	p.Asp51Gly	mis	c.749T>C	p.Leu250Pro	mis	Chinese	EOSRD	[91]
compHet	c.152A>G	p.Asp51Gly	mis	c.826G>T	p.Glu276*	nons	Chinese	EOSRD	[91]
compHet	c.152A>G	p.Asp51Gly	mis	c.733_735delGAG	p.Glu245del	indel	Chinese	LCA	[121]
n/s	c.157C>T	p.Arg53Trp	mis	n/s	n/s	n/s	n/s	LCA	[18]
compHet	c.190G>A	p.Gly64Arg	mis	c.834G>A	p.Trp278*	nons	White	LCA	[88]
compHet	c.190G>A	p.Gly64Arg	mis	c.834G>A	p.Trp278*	nons	n/s	LCA	[128]
compHet	c.190G>A	p.Gly64Arg	mis	c.834G>A	p.Trp278*	nons	British Caucasian	EORD	[134]
hom	c.211G>T	p.Val71Phe	mis	c.211G>T	p.Val71Phe	mis	n/s	n/s	[85]
							North African Jewish	LCA	[87]
							Israeli		[135]
							n/s		[153]
n/s				n/s	n/s	n/s	n/s		[1]
compHet	c.211G>T	p.Val71Phe	mis	c.216G>A	p.Trp72*	nons	n/s		[128]
compHet			mis			nons	Mixed	LCA	[87]
compHet	c.211G>T	p.Val71Phe	mis	c.733_735delGAG	[p.Glu245del]	indel	Chinese	LCA	[91]
compHet	[c.236T>C]	p.Met79Thr	mis	[c.834G>A]	p.Trp278*	nons	n/s	LCA	[125]
compHet			mis			nons	n/s	n/s	[85]
n/s	c.214T>C	p.Trp72Arg	mis	n/s	n/s	n/s	n/s	LCA	[1]
n/s				n/s	n/s	n/s	n/s	LCA	[18]
hom	c.215G>C	p.Trp72Ser	mis	c.215G>C	p.Trp72Ser	mis	n/s	LCA	[1]
compHet	c.214T>C	p.Trp72Arg	mis	c.265T>C	p.Cys89Arg	mis	Mixed	LCA	[87]
							n/s		[18]
compHet	c.221T>C	[p.Ile74Thr]	mis	c.616A>G	p.Ile206Val	mis	Chinese	EOSRD	[91]
compHet	c.224T>G	[p.Leu75Arg]	mis	c.421C>T	p.Gln141*	nons	Chinese	LCA	[91]
hom	c.236T>C	p.Met79Thr	mis	c.236T>C	p.Met79Thr	mis	Indian	LCA	[28,40]
compHet	c.237G>A	[p.Met79Ile]	mis	c.421C>T	p.Gln141*	nons	Chinese	LCA	[91]
hom	c.241C>T	p.Gly81*	nons	c.241C>T	p.Gly81*	nons	Chinese	LCA	[136]
n/s	c.244C>T	p.His82Tyr	mis	n/s	n/s	n/s	n/s	LCA	[1]
compHet	c.244C>T	p.His82Tyr	mis	c.286G>C	p.Asp90His	mis	n/s	LCA	[39,154]
hom	c.247G>A	p.Glu83Lys	mis	c.247G>A	p.Glu83Lys	mis	Indian	LCA	[155]
compHet	c.247G>A	p.Glu83Lys	mis	c.421C>T	p.Gln141*	nons	Chinese	LCA	[137]
hom	c.264G>A	p.Trp88*	nons	c.264G>A	p.Trp88*	nons	Pakistani	LCA	[88]
							n/s		[128]
							Bangladeshi		[28,40]
hom	c.265T>C	p.Cys89Arg	mis	c.265T>C	p.Cys89Arg	mis	n/s	LCA	[18,128]
							Spanish	LCA	[138]
n/s				n/s	n/s	n/s	n/s	LCA	[1]
compHet	c.265T>C	p.Cys89Arg	mis	c.618_619dupCT	p.Cys207Ser-fs*3	fs	n/s	LCA	[12]
hom	c.266G>A	p.Cys89Tyr	mis	c.266G>A	p.Cys89Tyr	mis	n/s	LCA	[89]
compHet	c.265T>C	p.Cys89Arg	mis	c.834G>A	p.Trp278*	nons	n/s	LCA	[128]
compHet	c.276+1G>A	[IVS2+1G>A]	splVar	c.834G>A	p.Trp278*	nons	n/s	LCA	[128]
compHet	c.276+6T>C	[IVS2+6T>C]	splVar	c.834G>A	p.Trp278*	nons	German	LCA	[139]
compHet	[c.(276+1_277-1)del]	n/s		c.815G>C	p.Arg272Pro	mis	n/s	LCA	[140]
het	c.277-2A>G	IVS2-2A>G	splVar	-	-	-	n/s	LCA	[125]
				-	-	-	n/s	n/s	[85]
n/s				n/s	n/s	n/s	n/s	LCA	[1]
compHet	c.277-2A>G	[in-frame ΔEx3]	splVar	c.784G>A	p.Gly262Ser	splVar	White	LCA	[88]
compHet	c.277-2A>G	[in-frame ΔEx3]	splVar	c.784G>A	p.Gly262Ser	splVar	n/s	LCA	[128]
compHet	c.277-2A>G	IVS2-2A>G	splVar	c.834G>A	p.Trp278*	nons	French	LCA	[28]
compHet							n/s		[128]
compHet							n/s	n/s	[85]
compHet							n/s	LCA	[153]
compHet							Australian	LCA	[9]
compHet				[c.834G>A]			Ireland, French	LCA	[40]
compHet							European		[87]
hom	c.286G>A	p.Val96Ile	mis	c.286G>A	p.Val96Ile	mis	Belgian	LCA	[40]
het				-	-	-	Portuguese	LCA	[28]
n/s				n/s	n/s	n/s	n/s	jRP	[41]
compHet	c.301T>C	[p.Ser101Pro]	mis	c.826G>T	p.Glu276*	nons	Chinese	LCA	[91]
compHet	c.325C>T	p.Gln109*	nons	c.421C>T	p.Q141*	mis	Chinese	LCA	[121]
het	c.341C>T	p.Thr114Ile	mis	-	-	-	n/s	LCA	[125]
				-	-	-		n/s	[85]
compHet	c.341C>T	p.Thr114I	mis	c.1126C>T	p.Pro376Ser	mis	U.S. American, French	LCA	[40]
							n/s	n/s	[85]
							n/s	LCA, LCA or EOSRD	[18]
							African American	LCA	[28]
n/s	c.341C>T, c.1126C>T (cis-allelic)	p.Thr114Ile, p.Pro376Ser	mis	n/s	n/s	n/s	African	LCA	[124]
				n/s	n/s	n/s	n/s	EOSRD	[18]
compHet	c.356_359del	p.His119Arg-fs*31	fs	c.834G>A	p.Trp278*	nons	Australian	LCA	[9]
hom	c.364G>A	p.Gly122Arg	mis	c.364G>A	p.Gly122Arg	mis	n/s	RP	[18]
compHet	c.364G>A	p.Gly122Arg	mis	c.421C>T	p.Gln141*	nons	Chinese	RP/LCA/ CORD	[92]
compHet	c.364G>A	p.Gly122Arg	mis	c.834G>A	p.Trp278*	nons	Italian	LCA	[93]
compHet	c.364G>C	p.Gly122Arg	mis	c.834G>A	p.Trp278*	nons	European	late-onset retinal degeneration	[87]
compHet							n/s	LCA, mild RP, RP	[18]
compHet	c.364G>A			c.834G>A			Italian	LCA	[93]
het	c.390C>A	p.His130Gln	mis	-	-	-	n/s	LCA or EOSRD (probably benign)	[88]
n/s				n/s	n/s	n/s	n/s	LCA or EOSRD	[18]
hom	c.401A>T	p.Tyr134Phe	mis	c.401A>T	p.Tyr134Phe	mis	French	LCA	[40]
het				-	-	-	Caucasian, Bangladeshi	LCA	[141]
compHet				c.1126C>T	p.Pro376Ser	mis	n/s	n/s	[142]
n/s	c.421C>T	p.Gln141*	nons	n/s	n/s	n/s	Chinese	LCA	[123]
hom				c.421C>T	p.Gln141*	nons	Turkish	LCA	[122]
							Chinese	LCA	[91,120,121,137]
								RP	[119]
compHet	c.421C>T	p.Gln141*	nons	c.433C>T	p.Gln145*	nons	Chinese	LCA	[91]
compHet	c.421C>T	p.Gln141*	nons	c.465+1G>A	[IVS3+1G>A]	splVar	Chinese	LCA	[91]
compHet	c.421C>T	p.Gln141*	nons	c.572T>C	p.Leu191Pro	mis	Chinese	CORD	[143]
compHet	c.421C>T	p.Gln141*	nons	c.572T>C	p.Leu191Pro	mis	Chinese	LCA	[121]
compHet	c.421C>T	p.Gln141*	nons	c.572T>C	p.Leu191Pro	mis	Chinese	EOSRD	[91]
compHet	c.421C>T	p.Gln141*	nons	c.581_584delACGA	p.Tyr194Trp-fs*14	fs	Chinese	LCA	[137]
compHet	c.421C>T	p.Gln141*	nons	c.602delA	p.Tyr201Ser-fs*7	fs	Chinese	LCA	[91]
compHet	c.421C>T	p.Gln141*	nons	c.646A>T	[p.Lys216*]	nons	Chinese	LCA	[91]
compHet	c.421C>T	p.Gln141*	nons	c.703_705del	p.Asn235del	indel	Chinese	LCA	[91]
compHet	c.421C>T	p.Gln141*	nons	c.834G>A	p.Trp278*	nons	Chinese	LCA	[121]
compHet	c.421C>T	p.Gln141*	nons	c.834G>A	p.Trp278*	nons	Chinese	LCA	[91]
compHet	c.421C>T	p.Gln141*	nons	c.834G>A	p.Trp278*	nons	Chinese	LCA	[121]
compHet	c.421C>T	p.Gln141*	nons	c.923T>C	p.Leu308Pro	mis	Chinese	CORD	[143]
hom	c.440T>C	p.Leu147Pro	mis	c.440T>C	p.Leu147Pro	mis	Chinese	LCA	[91]
hom	c.465G>T	p.His93_Gln155del (ΔEx3)	splVar	c.465G>T	p.His93_Gln155del (ΔEx3)	splVar	Pakistani	n/s	[126]
hom	c.465G>T	p.Gln155His	splVar	c.465G>T	p.Gln155His	splVar	Pakistani	LCA	[127]
compHet	c.465G>T	p.Gln155His	splVar	c.834G>A	p.Trp278*	nons	n/s	LCA	[140]
n/s	c.465+1G>A	IVS3+1 (IVS3+1G>A)	splVar	n/s	n/s	n/s	n/s	LCA	[1]
hom	c.465+1G>C	IVS3+1 (IVS3+1G>C)	splVar	c.465+1G>C	IVS3+1 (IVS3+1G>C)	splVar	n/s	LCA	[12]
het	c.466-2A>G	IVS3-2A>G	splVar	-	-	-	n/s	LCA	[141]
compHet	c.466-1G>C	[IVS3-1G>C]	splVar	c.834G>A	p.Trp278*	nons	n/s	LCA	[144]
hom	c.487C>T	p.Gln163*	nons	c.487C>T	p.Gln163*	nons	Middle Eastern	LCA	[88]
							Palestinian		[28,40]
									[145]
							n/s		[1,128]
							Emirati		[134]
compHet	c.517G>A	[p.Gly173Lys]	mis	c.572T>C	p.Leu191Pro	mis	Chinese	EOSRD	[91]
hom	c.547G>T	p.Gly183*	nons	c.547G>T	p.Gly183*	nons	n/s	LCA	[1]
compHet	c.554delG	[p.Gly187Glu-fs*23]	fs	c.642G>C	p.Val156_Lys214del (ΔEx4)	splVar	Chinese	LCA	[91]
compHet	c.572T>C	p.Leu191Pro	mis	c.642G>C	p.Val156_Lys214del (ΔEx4)	splVar	Chinese	EOSRD	[91]
compHet	c.582C>G	p.Tyr194*	nons	c.834G>A	p.Trp278*	nons	n/s	LCA	[18,128]
hom	c.589G>C	p.Ala197Pro	mis	c.589G>C	p.Ala197Pro	mis	Moroccan	LCA	[28,40]
							North African		[153]
het	c.593C>T	p.Ser198Phe	mis	-	-	-	n/s	LCA or EOSRD (probably benign)	[88]
n/s				n/s	n/s	n/s	n/s	LCA or EOSRD	[18]
n/s	c.617T>A	p.Ile206Asn	mis	n/s	n/s	n/s	n/s	LCA	[18]
het	c.641A>G	p.Lys214Arg	mis	-	-	-	n/s	LCA or EOSRD	[141]
compHet	c.643-2A>G	[p.IVS4-2A>G]		c.741T>A	[p.Tyr247*]	nons	Chinese	LCA	[91]
compHet	c.666G>A	p.Trp222*	nons	c.834G>A	p.Trp278*	nons	n/s	LCA	[18]
het	c.672insC	[p.Lys224Asn-fs]	fs	-	-	-	n/s	LCA	[141]
hom	c.689A>G	p.Asn230Ser	mis	c.689A>G	p.Asn230Ser	mis	Indian	LCA	[155]
hom	c.715T>C	p.Cys239Arg	mis	c.715T>C	p.Cys239Arg	mis	U.S. American	LCA	[22,40]
							n/s		[1]
n/s	c.723_725del	p.Leu241del3	indel	n/s	n/s	n/s	n/s	LCA	[1]
compHet	c.723_725del	p.Leu241del		[c.834G>A]	p.Trp278*	nons	European	LCA	[87]
hom	c.733_735del	p.Glu245del	indel	c.733_735del	p.Glu245del	indel	n/s	LCA	[140]
compHet	c.733G>T	p.Glu245*	nons	c.834G>A	p.Trp278*	nons	n/s	LCA	[18,128]
compHet	n/s	p.Leu257del9bp		[c.834G>A]	p.Trp278*	nons	U.S. American	LCA	[40]
hom	c.773G>C	p.Arg258Pro	mis	c.773G>C	p.Arg258Pro	mis	Pakistani	n/s	[126]
n/s	c.785-10delCTCCCCACAGGC	IVS5-10delCTCCCCACAGGC	splVar	n/s	n/s	n/s	n/s	LCA	[1]
n/s	c.784G>A	p.Gly262Ser	splVar	n/s	n/s	n/s	n/s	LCA	[1]
compHet	c.784G>A	p.Gly262Ser	splVar	c.834G>A	p.Trp278*	nons	n/s	LCA	[28]
							U.S. American		[40]
							European		[87]
compHet	c.809G>A	p.Arg270His	mis	c.834G>A	p.Trp278*	nons	Italian	LCA	[93,146]
compHet	c.809G>A	p.Arg270His	mis	c.834G>A	p.Trp278*	nons	n/s	LCA	[18]
compHet	c.809G>A	p.Arg270His	mis	c.834G>A	p.Trp278*	nons	n/s	LCA	[89]
monoallelic or compHet	c.815G>C	p.Arg272Pro	mis	n/s	n/s	n/s	Danish	LCA	[147]
hom	c.834G>A	p.Trp278*	nons	c.834G>A	p.Trp278*	nons	German	LCA	[131]
							African, White		[88]
							Saudi Arabian, U.S. American, Pakistani, Portuguese, Belgian		[40]
							n/s		[12,125,128,142,144,148]
							Pakistani		[22]
									[149]
									[150]
							Belgian		[124]
							Danish		[147]
							Spanish, French	LCA, RP, RD	[28]
								LCA	[153]
							Caucasian	LCA	[94]
							Northwestern European	LCA	[159]
							Italian	LCA	[93,146]
hom, monoallelic or compHet							n/s	LCA	[89]
n/s				n/s	n/s	n/s	n/s	LCA	[1]
n/s				n/s	n/s	n/s	Danish	LCA	[147]
het				-	-	-	n/s	n/s	[85]
compHet	[c.834G>A]	p.Trp278*	nons	n/s	p.Ala336del2bp	fs	U.S. American	LCA	[40]
			nons			fs	n/s	LCA	[22]
n/s	insGAGGCC	p.Glu280-ins6	indel	n/s	n/s	n/s	n/s	LCA	[1]
hom	c.844G>T	p.Glu282*	nons	c.844G>T	p.Glu282*	nons	Indian	LCA	[155]
hom	c.844_849dup	p.Glu282_Ala283-dup	indel	c.844_849dup	p.Glu282_Ala283-dup	indel	n/s	LCA	[128]
het	c.853G>A/c.854C>A	p.Arg285Gln	mis	-	-	-	n/s	LCA	[141]
hom	c.857A>T	p.Asp286Val	mis	c.857A>T	p.Asp286Val	mis	German	LCA	[139]
hom	c.862C>T	p.Gln288*	nons	c.862C>T	p.Gln288*	nons	Turkish	LCA	[151]
n/s	c.878T>C	p.Leu293Pro	mis	n/s	n/s	n/s	n/s	LCA	[1]
n/s				n/s	n/s	n/s	n/s	LCA	[18]
het	c.894G>C	p.Gln298His	mis	-	-	-	n/s	LCA or EOSRD	[88]
n/s				n/s	n/s	n/s	n/s	LCA or EOSRD	[18]
hom	c.905G>T	p.Arg302Leu	mis	c.905G>T	p.Arg302Leu	mis	Indian	LCA	[28,40,145]
hom, het							Indian, Pakistani, Iranian	LCA or EOSRD, unaffected	[88]
het				-	-	-	n/s	n/s	[85]
				-	-	-	Italian	LCA	[146]
				-	-	-	n/s	n/s	[142]
				n/s	n/s	n/s	n/s	LCA	[89]
hom	c.910G>T	p.Glu304*	nons	c.910G>T	p.Glu304*	nons	Indian	LCA	[155]
hom	c.926_927insCCTGAACCGCAGGGAGCT	p.Glu309Asp-insLNRREL	indel	c.926_927insCCTGAACCGCAGGGAGCT	p.Glu309Asp-insLNRREL	indel	Chinese	LCA	[90]
							n/s	LCA	[18]
het	c.971G>T	p.Arg324Leu	mis	-	-	-	n/s	LCA	[89]
hom	c.1003insG	[p.Pro335Ala-fs]	fs	c.1003insG	[p.Pro335Ala-fs]	fs	n/s	LCA	[141]
monoallelic het	c.1053_1064delTGCAGAGCCACC	p.Ala352_Pro355del (also known as. p.Pro351-del12bp)	indel	-	-	-	n/s	adCORD, juvenile RP	[18,28]
het	c.1076C>T	p.Ser359Phe	mis	-	-	-	n/s	LCA	[141]
n/s	c.1091C>G	p.Ala364Gly	mis	n/s	n/s	n/s	n/s	LCA or EOSRD (probably benign)	[18]
het	c.1097C>G	p.Pro366Arg	mis	-	-	-	n/s	LCA or EOSRD (probably benign)	[88]
n/s				n/s	n/s	n/s	n/s	LCA or EOSRD	[18]
n/s	insCAGAGCCAGCCA	p.Ala368-ins12	indel	n/s	n/s	n/s	n/s	LCA	[1]
n/s	c.1103_1114dup	p.Glu369_Thr372-dup	indel	n/s	n/s	n/s	n/s	LCA	[18]
het	c.1111_1122dup	p.Ala371_Pro374-dup	indel	-	-	-	n/s	LCA	[125]
n/s				n/s	n/s	n/s	n/s	LCA	[18]
het	c.1126C>T	p.Pro376Ser	mis	-	-	-	West African, African American, Caribbean	LCA (probably benign)	[88]
				-	-	-	n/s	n/s	[142]
hom				c.1126C>T	p.Pro376Ser	mis	West African, African American, Caribbean	LCA or EOSRD (probably benign)	[88]
n/s				n/s	n/s	n/s	n/s	LCA or EOSRD (probably benign)	[18]

compHet—compound heterozygous; hom—homozygous; het—heterozygous; mis—missense; nons—nonsense; fs—frameshift; indel—in-frame indel; splVar—slice variant; n/s—not specified; -—no second allele was identified; asterisks (*) indicate the occurrence of a stop codon; square brackets mean mutation was not stated directly in the reference but was deduced. Cohorts are indicated according to the references.

**Table 3:** AAV-*AIPL1* vector.

Therapy	Model	Outcomes	Reference
AAV2/2-CMV-*hAIPL1*-SV40, AAV2/8-CMV-*hAIPL1*-SV40, AAV2/2-CMV-*mAipl1*-SV40, AAV2/8-CMV-*mAipl1*-SV40	AIPL1 null and hypomorphic mouse	increased production of AIPL1 in the photoreceptor inner segment, increased levels of PDE6 in the outer segment, slowdown of retinal degeneration, improved photoreceptor cell survival, preservation of outer segment morphology, stabilization of retinal function	[48]
scAAV-Y733F-RKp-*hAipl1*	AIPL1 null mouse	scAAV variant induces earlier and higher expression of hAIPL1 compared to ssAAV, restoration of rod and cone PDE6 expression, and slowdown of photoreceptor degeneration, preservation of photoreceptor ultrastructure, functional vision rescue	[176]
scAAV2/8-Y733F-pRK-*hAIPL1*	adCORD mouse	rescue of photopic cone-mediated ERG responses, improvement of visual acuity in photopic conditions, scotopic rod-mediated ERG responses did not improve	[66]
AAV9-*AIPL1wt*, AAV9-*AIPL1co*	ARPE-19 cells	AAV9-AIPL1co induced significantly less antiviral response compared to wtAIPL1	[189]
rAAV2.7m8.hRK.*AIPL1*	organoids	restored AIPL1 protein abundance, rescue of rod PDE6, and cGMP levels	[190]
rAAV8.hRKp.*AIPL1*	patients	improvement of visual acuity, enhanced activity of the specific to the treated eye’s visual cortex, better preservation of retinal thickness and structural lamination of the outer retina in the treated eye than in the untreated eye, cystoid macular edema (treated eye of only one out of four patients)	[12]

## Data Availability

No new data were created or analyzed in this study. Data sharing is not applicable in this article.

## Supplementary Materials

The following supporting information can be downloaded at: https://www.mdpi.com/article/10.3390/ijms262412066/s1.

## Abbreviations

The following abbreviations are used in this manuscript:

AIPL1	Aryl hydrocarbon receptor interacting protein-like 1
AIP	Aryl hydrocarbon receptor interacting protein
AhR	Aryl hydrocarbon receptor
LCA	Leber congenital amaurosis
LCA4	Leber congenital amaurosis type 4
EOSRD	Early-onset severe retinal dystrophy
RP	Retinitis pigmentosa
IRD	Inherited retinal diseases
RPE	Retinal pigment epithelium
ONL	Outer nuclear layer
AAV	Adeno-associated virus
FKBP	FK506-binding protein
TPR	Tetratricopeptide repeat
PRD	Proline-rich domain
HSP70	70 kilodalton heat shock proteins
HSP90	90 kilodalton heat shock proteins
PDE6	Phosphodiesterase 6
PDE6α	Phosphodiesterase 6 subunit α
PDE6α′	Phosphodiesterase 6 subunit α′
PDE6β	Phosphodiesterase 6 subunit β
PDE6γ	Phosphodiesterase 6 subunit γ
PDE6γ’	Phosphodiesterase 6 subunit γ’
Pγ	Phosphodiesterase 6 subunit γ
Pγ’	Phosphodiesterase 6 subunit γ
NUB1	NEDD8 ultimate buster 1
FAT10	Human leukocyte antigen F adjacent transcript 10
CDS	Coding sequence
RT-PCR	Reverse transcription polymerase chain reaction
OCT	Optical coherence tomography
ERG	Electroretinography
iPSC	Induced pluripotent stem cell
UTR	Untranslated region
cryo-EM	Cryogenic electron microscopy
NMR	Nuclear magnetic resonance
BLI	Bio-layer interferometry
SPR	Surface plasmon resonance
ELISA	Enzyme-linked immunosorbent assay
